# Peptide Activator Stabilizes DJ-1 Structure and Enhances Its Activity

**DOI:** 10.3390/ijms252011075

**Published:** 2024-10-15

**Authors:** Jing-Yuan Shih, Yuan-Hao Howard Hsu

**Affiliations:** Department of Chemistry, Tunghai University, No. 1727, Sec. 4, Taiwan Boulevard, Xitun District, Taichung 40704, Taiwan; rrichaard1998@gmail.com

**Keywords:** Parkinson’s disease, DJ-1, hydrogen/deuterium mass spectrometry, *PARK7*

## Abstract

DJ-1 is a vital enzyme involved in the maintenance of mitochondrial health, and its mutation has been associated with an increased risk of Parkinson’s disease (PD). Effective regulation of DJ-1 activity is essential for the well-being of mitochondria, and DJ-1 is thus a potential target for PD drug development. In this study, two peptides (^15^EEMETIIPVDVMRRA^29^ and ^47^SRDVVICPDA^56^) were utilized with the aim of enhancing the activity of DJ-1. The mechanisms underlying the activity enhancement by these two peptides were investigated using hydrogen/deuterium exchange mass spectrometry (HDXMS). The HDXMS results revealed distinct mechanisms. Peptide 1 obstructs the access of solvent to the dimer interface and stabilizes the α/β hydrolase structure, facilitating substrate binding to a stabilized active site. Conversely, peptide 2 induces a destabilization of the α/β hydrolase core, enhancing substrate accessibility and subsequently increasing DJ-1 activity. The binding of these two peptides optimizes the activity site within the dimeric structure. These findings offer valuable insights into the mechanisms underlying the activity enhancement of DJ-1 by the two peptides, potentially aiding the development of new drugs that can enhance the activity of DJ-1 and, consequently, advance PD treatment.

## 1. Introduction

Parkinson’s disease is a neurodegenerative disease, mainly affecting the central nervous system. In patients with Parkinson’s disease (PD), α-synuclein protein aggregates and dopamine-generating cells die in the substantia nigra [1]. Although the exact cause of PD remains unclear, environmental toxins and genetic disorders are recognized as two risk factors for the disease. Both factors can lead to mitochondrial dysfunction, initiating neuronal cell death and triggering PD [2,3]. Dysregulated mitochondria contribute to the overproduction of reactive oxygen species (ROS), significantly impairing mitochondrial function and reducing the efficiency of the electron transport chain and ATP production, particularly in electron transport chains I and III [4]. Mutations in α-synuclein [5], Parkin (*PARK2*) [6], PINK1 (*PARK6*) [7], DJ-1 (*PARK7*) [8], and iPLA_2_β (*PARK14*) [9] have been identified as being associated with mitochondrial impairment and the underlying causes of PD.

**DJ-1 is a Potential Drug Target for PD**

DJ-1, a deglycase encoded by *PARK7* [10], has been associated with PD, with the association due to the loss of its enzymatic activity and its ability to interact with other proteins [8]. DJ-1 forms complexes with Parkin and PINK1 to repair mitochondrial function in response to oxidative stress [11]. Both Parkin and PINK1 exhibit neuroprotective activity against oxidative stress [12,13]. PINK1, also known as PTEN-induced putative kinase, localizes in mitochondria to activate Parkin [14]. Together, these three proteins collaborate to regulate ROS in neuronal cells and maintain mitochondrial functionality. Furthermore, DJ-1 protein was demonstrated to directly coaggregate with α-synuclein through hydrophobic interactions, suggesting a potential connection to the initiation of α-synuclein aggregation [15]. Moreover, DJ-1 was shown to play a role in the transcriptional regulation of dopamine synthesis [16] and to interact with a large protein complex involved in the regulation of catecholamine homeostasis [17]. Dysfunction of DJ-1 may lead to dopamine oxidation, contributing to the aggregation of α-synuclein [18,19,20,21,22]. This implies that the oxidation of dopamine by ROS causes the precipitation of α-synuclein in the substantia nigra.

The endogenous antioxidant DJ-1 exhibits redox-activated chaperone activity and functions as a cytoprotective agent [23,24,25]. DJ-1 can interact with NADPH oxidase, thereby regulating ROS production in macrophages [26]. Additionally, DJ-1 may upregulate glutathione synthesis to counteract α-synuclein toxicity [27]. DJ-1 was demonstrated to have specific protective effects on astrocytes against rotenone-induced neurotoxicity in a rat model of PD [28]. DJ-1 has become a potential drug target for PD. The small molecules, compound **23** [29], compound **B** [30] and the ND-13 peptide [31] have been shown to enhance the cellular functions through DJ-1 activity.

**Structure and Cellular Function of DJ-1**

DJ-1, a cytosolic protein containing 189 amino acids and with a molecular weight of 20 kDa, can localize to mitochondria in response to oxidative stress [32]. Through cysteine palmitoylation, DJ-1 can also localize to lipid rafts, thereby regulating endocytosis in astrocytes [33]. DJ-1 exhibits multiple enzymatic activities, including deglycase activity [34], chaperone activity [35], proteolytic activity [36], and glyoxalase activity [37]. Of the three cysteine residues in DJ-1 (Cys46, Cys53, and Cys106), Cys106 serves as the active site [38]. The antioxidative defense mechanism has been associated with the oxidation of Cys106 into sulfinic acid [38]. Both the crystal structure of DJ-1 and various cellular results support the understanding that DJ-1 exists in dimeric form [39,40]. Mutations such as L166P and M26I on the interaction interface hinder DJ-1 activity, underscoring the crucial role of the dimer structure in its functionality [41].

Because of the observed decrease in DJ-1 activity resulting from Cys106 oxidation and mutation variants, our intention was to design peptides that can interact with the dimer interface, thereby enhancing the enzymatic activities of DJ-1. The effects of peptide binding and the mechanisms underlying activity enhancement were comprehensively analyzed using hydrogen/deuterium exchange mass spectrometry (HDXMS).

## 2. Results

### 2.1. Peptide Design

**Figure 1:**
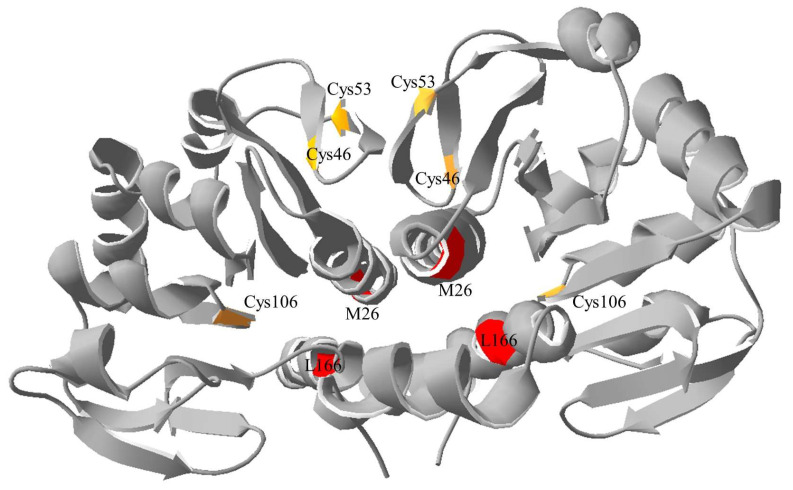
The dimerization configuration of DJ-1. The crystal structure of DJ-1 (PDB: 2OR3) showing the three cysteine residues in yellow and two critical residues in red related to the dimerization of DJ-1.

In pursuit of therapeutic outcomes for PD, Daniel Offen and colleagues designed multiple peptides derived from the primary sequence of DJ-1. These peptides could protect cells against oxidative and neurotoxic damage, thereby reducing intracellular ROS accumulation [31]. One such peptide, ND-13 (^12^KGAEEMETVIPVD^24^), was also demonstrated to exhibit cardioprotective efficacy [42]. However, the mechanisms underlying these cellular effects remain unclear. Because the active dimeric structure of DJ-1 influences its activity, DJ-1’s dimeric nature can be effectively disrupted by binding peptides to it to alter its functionality. The dimerization configuration of DJ-1 was shown by the crystal structure (Figure 1). There are three cysteine residues on DJ-1, including the active site Cys106 and two critical residues, Met26 and Lys166, for the dimerization of DJ-1. An analysis of the crystal structure of DJ-1 (PDB: 2OR3) revealed that the dimerization interface comprises an α-helix segment (Glu15–Ala29) and a β-sheet segment (Ser47–Ala56). In the α-helix contact region (Glu15–Ala29), the interaction between Glu15 and Arg28 takes the form of intermolecular hydrogen bonds and salt bridges. Hydrophobic amino acids—such as Ile, Pro, Ala, Met, and Val—contribute to hydrophobic forces at the binding site, facilitating dimerization. To preserve the α-helix structure, the designed short peptide, named peptide 1 (P1; Glu15–Ala29), includes the entire α-helix.

At the contact interface of the β-sheet (Ser47–Ala56), hydrogen bonds can form between Val51 and Cys53 as well as between Arg28 and Arg48 due to their close proximity. These hydrophobic interactions and hydrogen bonds contribute to the stability of the dimeric structure. To preserve the β-sheet secondary structure, the designed peptide, named peptide 2 (P2; Ser47–Ala56), includes the entire β-sheet. In experiments involving hydrogen–deuterium exchange, the sequence of P1 was observed to overlap with the pepsin-digested sequence of DJ-1. To comprehensively analyze hydrogen–deuterium (H/D) exchange, Val20 was substituted with the structurally similar amino acid Ile. Two purchased peptide segments, namely P1 (V20I) and P2, were employed, and their interactions with DJ-1 were investigated.

### 2.2. DJ-1 Purification and Activity Measurement

**Figure 2:**
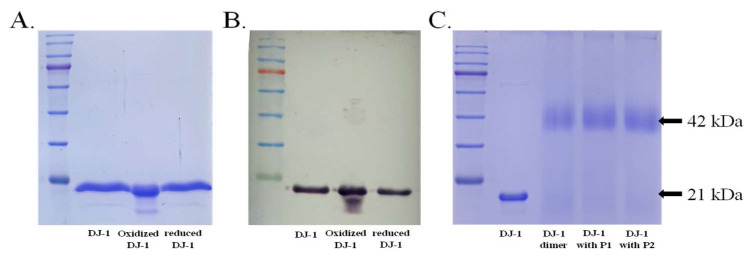
Purification and verification of DJ-1. (**A**) SDS-PAGE analysis of DJ-1, stained with Coomassie blue, revealing distinct bands at approximately 21 kDa. From left to right, the samples are DJ-1, the oxidized form of DJ-1 with two bands, and DTT-reduced DJ-1. (**B**) Western blot analysis by using anti-DJ-1 as the primary antibody revealed clearly defined bands at approximately 21 kDa. The samples are DJ-1, the oxidized form of DJ-1, and reduced DJ-1. (**C**) DJ-1 was treated with the DSS cross-linker for SDS-PAGE. Following DSS cross-linking, prominent dimer bands were observed at 42 kDa. The samples, from left to right, are DJ-1, DSS-crosslinked DJ-1, and DSS-crosslinked DJ-1 following the addition of P1 and P2.

The DJ-1 protein with the 6X-His tag was overexpressed using pET3a-His-DJ1-transformed *Escherichia coli* and subsequently purified using Ni–nitrilotriacetic acid agarose beads. The sodium dodecyl sulfate–polyacrylamide gel electrophoresis (SDS-PAGE) patterns of eluted DJ-1 had either one or two bands across different fractions (Figure 2A). Western blot analysis confirmed both bands as corresponding to DJ-1 (Figure 2B). Following a 1 M DTT reduction of the sample with double bands, the SDS-PAGE pattern contained a single band. This confirmed the lower band as the oxidized form of DJ-1. The deglycation activity of the purified DJ-1 was then assessed. A substrate was prepared through the reaction of 10 mM *N*-acetyl-L-cysteine with 10 mM methylglyoxal solution. After 10 min of reaction, the reaction plateaued. DJ-1 demonstrated the deglycase activity to glyoxal-glycated cysteine, as indicated by the reduction in UV absorbance at 288 nm.

The active form of DJ-1 has been demonstrated to form a homodimer conformation in its crystal structure. The purified DJ-1 was confirmed to exhibit the dimeric conformation through DSS cross linking (Figure 2C). Two peptides, designated P1 and P2, were specifically designed to disrupt the dimerization interfaces of DJ-1 based on its crystal structure. The designed peptides, P1 and P2, were found to enhance the activity of DJ-1, as indicated by the MGO activity assay. Upon peptide binding, further analysis of DJ-1 dimerization was conducted using DSS cross linking and SDS-PAGE. The results of both indicated that peptide binding did not disrupt the dimer structure of DJ-1 (Figure 2C).

**Figure 3:**
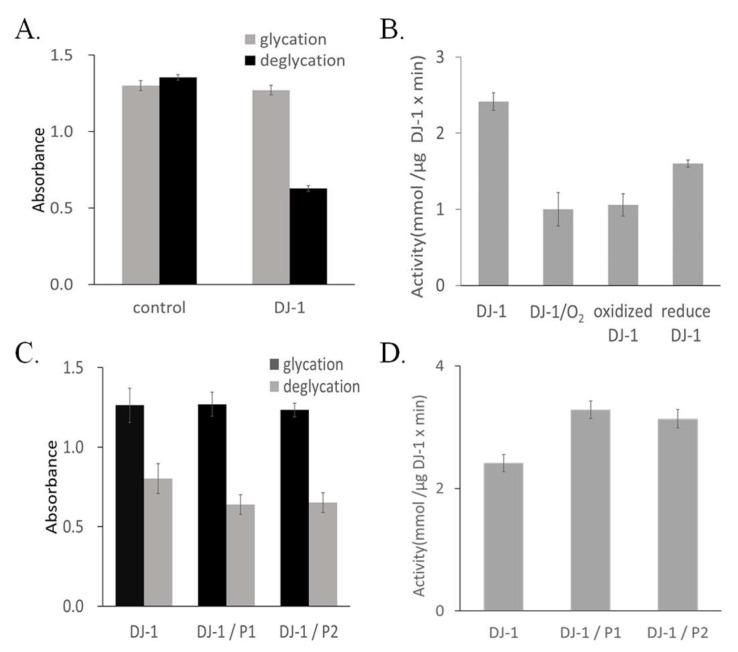
DJ-1 activity measurement. (**A**) The deglycation assay of DJ-1 involved the breakdown of hemithioacetal and resulted in a significant decrease in absorbance. The control corresponds to no DJ-1 protein. (**B**) The activity of DJ-1 was measured using a deglycation assay. The samples included were degassed assay DJ-1, DJ-1 without degassed assay, oxidized DJ-1, and reduced DJ-1; variation was discovered in deglycation activity under different conditions. (**C**) DJ-1 with or without P1 and P2 was added to the prepared substrate, and the resultant mixture was incubated at 37 °C for 30 min. (**D**) Calculated activities from (**C**).

Under anaerobic conditions, DJ-1 broke down glyoxal-glycated cysteine, resulting in a reduction in UV absorbance, thus confirming the deglycase activity of DJ-1 (Figure 3A). The activity was calculated to be 2.25 mmol/µg DJ-1/min. However, under aerobic conditions, the activity of DJ-1 was much lower at 1.00 mmol/µg/min; this prompted the assay to be conducted in a deoxygenated solution (Figure 3B). The oxidized form of DJ-1, characterized by two bands on SDS-PAGE patterns, exhibited activity of 1.06 mmol/µg/min. Following DTT reduction of DJ-1, its activity was restored to 1.60 mmol/µg/min. These findings indicate that oxidation reduces the activity of DJ-1 and that DTT reduction of oxidized DJ-1 can partially restore its functionality. The addition of P1 resulted in a notable 36% increase in the activity of DJ-1, which reached 3.28 mmol/µg/min. Similarly, the addition of P2 led to a 30% improvement in the activity of DJ-1, which reached 3.14 mmol/µg/min (Figure 3C,D).

### 2.3. Identification of Proteolyzed DJ-1 Fragments

**Figure 4:**
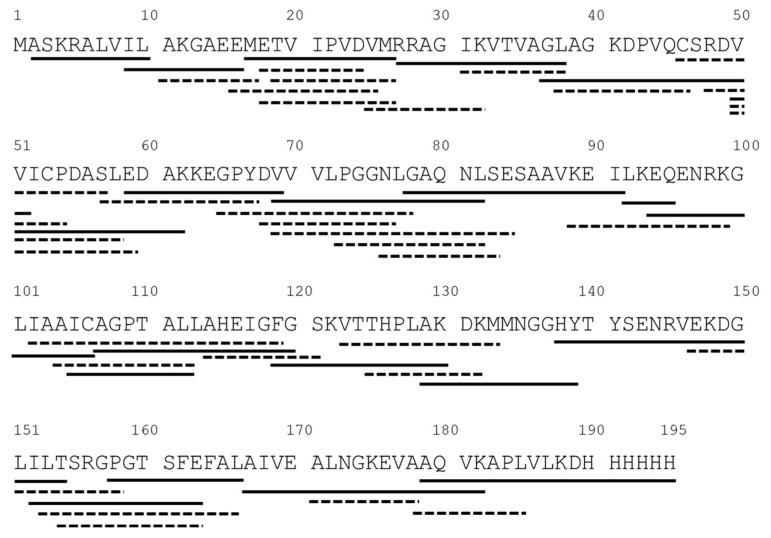
Peptide map of DJ-1 protein. DJ-1 protein was digested using a self-packed pepsin column on ice, and the resulting peptic fragments were retained on a peptide trap. Subsequently, the peptides were eluted through a C18 column by using a gradient for mass spectrometry analysis. The MS/MS data were exported to X! Tandem for sequence identification. The solid and the dashed lines are the identified peptides and the solid lines are selected for further structural presentation.

Purified DJ-1 protein was obtained and validated using SDS-PAGE and Western blotting. The activity of DJ-1 was subsequently confirmed through the deglycase activity assay. The protein was then injected into a high-performance liquid chromatography system with a pepsin digestion column and a C18 reverse-phase column, and ion-trap mass spectrometry was then performed. The DJ-1 was digested by pepsin, and the digested peptides of DJ-1 were separated using a C18 column. The eluted peptides were then subject to tandem mass spectroscopy (MS/MS) by using ion-trap mass spectrometry, and the sequences of the fragments were identified using the X! Tandem-parser-1.7.7 program. The identified fragments were mapped onto the primary sequence of DJ-1 (Figure 4). In total, 49 peptides were identified, and these 49 covered 99.5% of the protein sequence (Supplemental Table S1). Notably, the initiator methionine was not identified in the map, which may be attributable to posttranslational excision. The His tag at the C-terminus was also identified.

### 2.4. H/D Analysis of DJ-1 and the Effects of Peptide Binding

**Figure 5:**
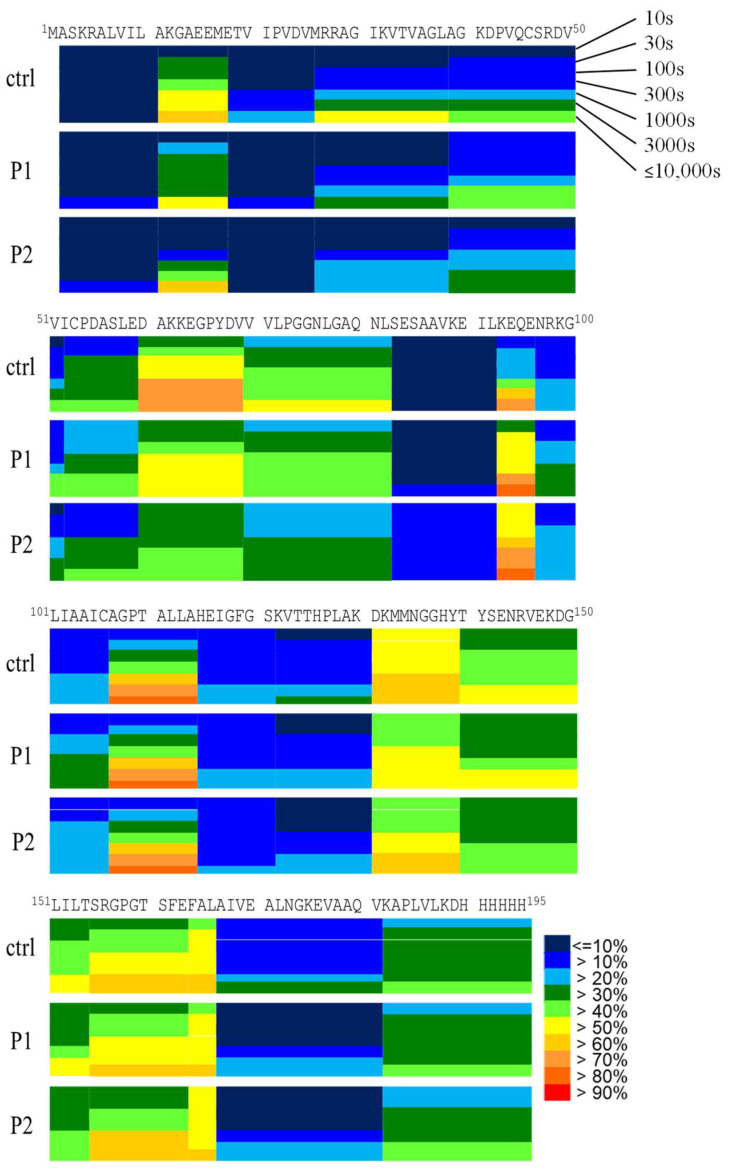
Deuteration levels of DJ-1. DJ-1 was incubated with or without P1 and P2 at 37 °C for 10 min, followed by deuteration in an H/D exchange reaction from 10 to 10,000 s. The reactions were quenched using ice-cold quench solution containing formic acid and guanidine hydrochloride. The deuterated protein was then subjected to mass spectrometry analysis. The number of deuterons incorporated in each peptide was measured, and the deuteration levels were subsequently calculated.

The HDXMS analysis of DJ-1 highlighted its solvent accessibility and the flexibility of its backbone. H/D exchange was performed on the DJ-1 protein for 10–10,000 s. To ensure the homogeneity of DJ-1, the protein samples were treated with 1 M DTT, and residual DTT was removed using a PALL centrifugal filtration device. The deuteration levels of each pepsin-digested peptide were quantified at seven time points (Supplemental Figure S1). Because deuteration can be back-exchanged during mass spectrometry analysis, the data were normalized based on the 24 h full exchanged data (Supplemental Figure S2). Although 48 h exchange experiments were also conducted, unfortunately, the protein was precipitated. The deuteration percentage levels were then calculated based on the maximal deuteration of each peptide, and representing peptides were selected and color-coded to illustrate the exchange levels (Figure 5). Peptides 17–25, 18–24, 18–26, and 19–26 exhibited the lowest H/D exchanging region, with approximately 40% deuteration after 10,000 s of exchange. By contrast, peptides 59–69, 73–82, 76–83, 138–154, 147–158, and 154–163 exhibited relatively rapid exchange, reaching approximately 65% deuteration after 10,000 s of exchange.

**Figure 6:**
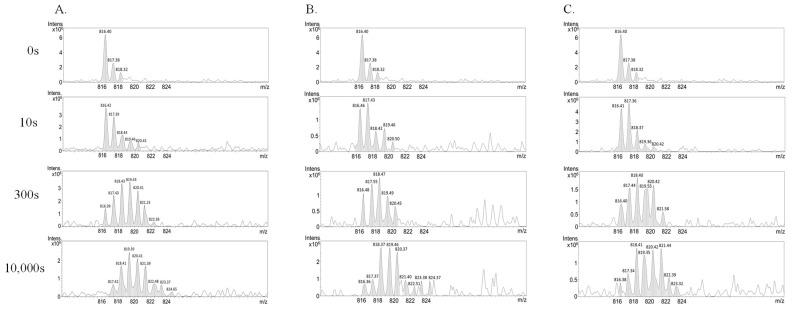
**H**/D exchange mass spectrum in the region 104–112 upon P1 and P2 binding. DJ-1 underwent deuteration from 0 to 10,000 s, and the results at 0, 10, 300, and 10,000 s are presented. (**A**) Mass spectrum of the sequence 104–112 after HDXMS. (**B**) HDXMS results of DJ-1 interacting with P1. (**C**) HDXMS results of DJ-1 interacting with P2.

The exposed active site on the α/β hydrolase core exhibited a higher level of deuteration when the period of H/D exchange was longer. The active site triad comprises Glu18, His126, and Cys106, with the oxidation status of Cys106 being linked to the activity of DJ-1. His126 was located in a domain exhibiting a low rate of H/D exchange, whereas Glu18 resided in a highly conserved acidic region, including Glu15, 16, and 18, and experienced a relatively high rate of H/D exchange in the N-terminus. Peptide 104–112 included the active site Cys106 in its sequence, making it ideal for representing the corresponding changes in the active site. The mass spectrum displayed in Figure 6A reveals a clear shift of the mass envelope, indicating an increase in deuteration over time. After 10,000 s of exchange, peptide 104–112 exhibited a mass increase of 4.4 Da. In the P1 and P2 addition experiments (Figure 6B,C), the mass shift further increased to 4.8 Da after P1 and P2 binding.

P1 or P2 was combined with DJ-1 in a molar ratio of 10:1 and allowed to react at 37 °C for 10 min. H/D exchange reactions were then conducted over a time range of 10–10,000 s. The changes in H/D exchange levels after P1 or P2 binding were assessed. Following the addition of P1 or P2, the exchange levels of most sequences were slightly lower. In the P1 experimental group, a significant reduction in H/D exchange was observed at 10,000 s for peptides 19–26 and 27–38. Critical regions for enzymatic activity—the active catalytic triad Glu18, His126, and Cys106—exhibited noteworthy decreases in H/D exchange. Peptides 17–25 and 9–16, containing Glu18, exhibited noticeable decreases, whereas peptide 123–133, containing His126 in its sequence, exhibited a decrease at 10,000 s. Notably, although the overall results revealed a reduction in H/D exchange, regions 78–91 and 92–96 demonstrated an increase. In the P2 experimental group, the trends in H/D exchange for most sequences were similar to those in the P1 experimental group. Peptides 19–26, 27–38, and 31–38 as well as peptides containing the active catalytic triad (peptides 17–26 and 123–133) exhibited varying degrees of decrease in H/D exchange. Furthermore, regions 78–91 and 92–96 demonstrated varying degrees of increase in H/D exchange. Notably, unlike the P1 experimental group, an increase in H/D exchange was noted in peptide 2–10 at 10,000 s.

**Figure 7:**
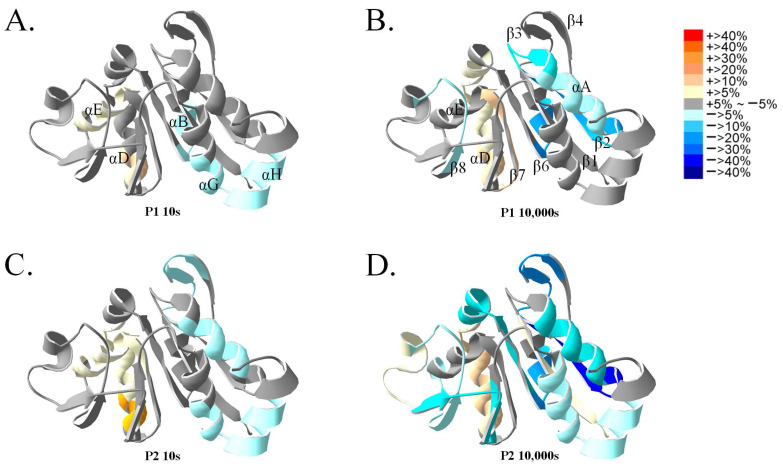
Effects of P1 and P2 peptide binding on DJ-1, as analyzed using HDXMS. DJ-1 protein was incubated with P1 and P2 peptides for 10 min. The changes in HDXMS levels at 10 s and 10,000 s were mapped onto the DJ-1 crystal structure (2OR3.PDB). The representations of P1 treatment at 10 s (**A**), P1 treatment at 10,000 s (**B**), P2 treatment at 10 s (**C**), and P2 treatment at 10,000 s (**D**) are shown to illustrate the changes in HDXMS levels.

To further examine the interaction of and structural changes in DJ-1 induced by P1 and P2, the differences in H/D exchange percentages between P1 and P2 adsorption and the control group were mapped onto the crystal structure (Figure 7). Following the addition of P1 or P2, at 10 s of H/D exchange, slight reductions in exchange were observed in the αG and αH-helices, whereas increases in exchange were noted in the αD and αE-helices. However, the overall change in H/D exchange at 10 s was limited. At 10,000 s of H/D exchange, both P1 and P2 exhibited evident reductions in exchange at the interface of the dimer in the αA-helix, β2, and β4 sheets, with a more pronounced decrease in the β2, β3, and β4 sheets in the P2 experimental group. Additionally, on the backside of the active site in the αD-helix, a significant increase in H/D exchange was observed in both the P1 and P2 experimental groups. By contrast, in the P2 experimental group, the β1-sheet exhibited an increase in H/D exchange, and the β5 sheet exhibited a decrease, whereas in the P1 experimental group, no notable change was observed in the β1 and β5 sheets.

### 2.5. H/D Exchange of Peptide 1 Activated DJ-1

**Figure 8:**
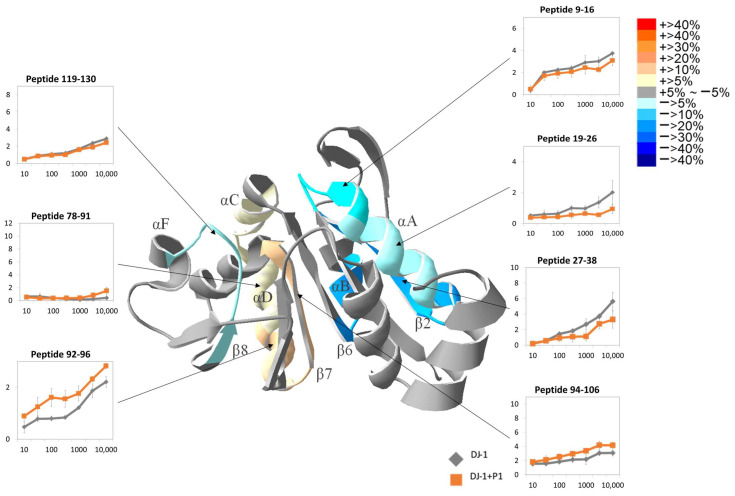
Changes in H/D exchange observed in HDXMS after binding of DJ-1 with peptide 1. DJ-1 bound to P1 was deuterated for 10, 30, 100, 300, 1000, 3000, and 10,000 s. The HDXMS results of the selected peptides are presented in the linear graph. The structural graph depicts the changes in each fragment at 10,000 s. The assorted colors in the heat map represent the various levels of changes. All experiments were conducted in triplicate, and the errors represent the standard deviation.

The addition of P1 resulted in 36% activation of the deglycation activity of DJ-1, indirectly confirming interactions between DJ-1 and peptide P1. However, the tertiary structure of peptide-bound DJ-1 remained the dimer conformation. Figure 8 presents the HDXMS results of selected peptides after the reaction of DJ-1 with P1, along with the 10,000 s-HDXMS mapped structure. The detailed HDXMS results of all peptides before and after back exchange correction are shown in Supplemental Figures S1 and S2. In peptide 94–106, which contains the active site Cys106, an increase in the deuteration level was observed, and near the dimer interface region—particularly in peptides 17–25, 17–26, 18–24, 18–26, and 19–26, assumed to be binding sites for P1—a trend of decreased H/D exchange was evident. The H/D exchange in peptide 19–26 exhibited decreased slightly starting at 30 s and reached the maximum decrease of 21.5% at 10,000 s. At peptide 27–38, located at the center of the dimer interface, the H/D exchange changed slightly starting from 100 s and reached the maximum decrease of 23.3% at 10,000 s. Peptide 31–38 in the same region also exhibited a trend of decreased H/D exchange. At 10,000 s, peptides 9–16 (−11.0%) and 119–130 (−5.2%) in the catalytic region exhibited decreased H/D exchange. A particularly noteworthy observation was that regarding the region opposite the active site, including peptides 78–91, 92–96, 94–106, and 102–119, because H/D exchange was increased at all of these. The H/D exchange at peptide 78–91 started to increase at 3000 s, reaching a 9% increase at 10,000 s. Peptide 92–96 exhibited the greatest increase in H/D exchange, with a 19.1% increase at 10 s and a 20.4% increase at 10,000 s. At peptide 94–106, the H/D exchange increased slightly starting from 30 s, with a 9.8% increase at 10,000 s. At peptide 102–119, the H/D exchange had increased by 15.8% at 10,000 s.

### 2.6. H/D Exchange in DJ-1 Activated by Peptide 2

**Figure 9:**
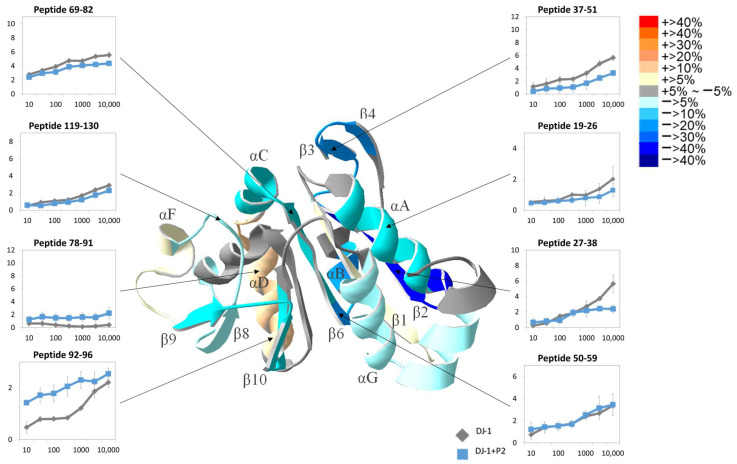
Changes in H/D exchange observed in HDXMS after binding of DJ-1 with peptide 2. DJ-1 bound to P2 was deuterated for 10, 30, 100, 300, 1000, 3000, and 10,000 s. The HDXMS results of the selected peptides are presented in the linear graph. The structural graph depicts the changes in each fragment at 10,000 s. The assorted colors in the heat map depict various levels of changes. All experiments were conducted in triplicate, and the errors represent the standard deviation.

The alterations in HDXMS after the reaction of DJ-1 with P2 are presented on the structure of DJ-1 in Figure 9. The detailed HDXMS results of all peptides before and after back exchange correction are shown in Supplemental Figures S3 and S4. Notably, a slight increase in H/D exchange was observed at peptides 78–91 and 92–96. By contrast, decreases in exchange were noted in peptide 19–26 at the dimer interface and peptide 119–130 at the positions of Glu18 and His126 in the catalytic triad. As the exchange time increased, the decrease in H/D exchange extended to the entire protein. In the DJ-1 dimeric interface region, speculated to be the site of the interaction with P2, a slight 5% decrease in H/D exchange was observed from 300 s in peptide 50–58, increasing to a 10.0% decrease at 10,000 s (Supplemental Figure S3). Similarly, the exchange at peptide 19–26, containing αA at the dimeric binding interface, exhibited a slight decrease from 7.2% at 300 s to 14.7% at 10,000 s. Peptide 37–51, in the middle of the binding interface, exhibited deuterium exchange decreases of 14.3% at 10 s and 13.1% at 10,000 s. Moreover, peptide 59–69 exhibited a 26.2% H/D exchange decrease at 10,000 s. In the catalytic region, peptide 119–130 exhibited a 6.7% decrease in deuterium exchange at 10,000 s. Conversely, peptides 78–91 and 92–96, located in the opposite direction to the active site, exhibited increases of 5.1% and 31.8% at 10 s, respectively, reaching 14.9% and 11.1% at 10,000 s. However, in the central β-sheet of the αβ-sandwich, the deuterium exchange differed from that observed in the P1 experimental group. In peptide 2–10 of the β1 sheet, a 7.8% increase in deuterium exchange was observed at 10,000 s, whereas in peptide 69–82 of the β6 sheet, a 10.9% decrease in deuterium exchange was observed at 10,000 s.

### 2.7. H/D Exchange in Nonreduced DJ-1

**Figure 10:**
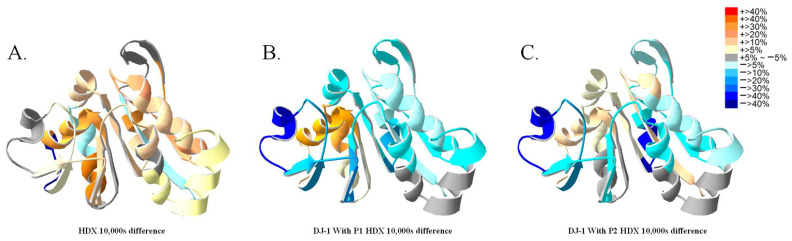
Peptide binding effects of the reduced DJ-1, as observed through HDXMS. (**A**) Differences in HDXMS results between the originally reduced DJ-1 and the DTT-reduced DJ-1. Peptides P1 (**B**) and P2 (**C**) were bound to the original DJ-1 without further reduction at 37 °C. The differences in deuteration levels at 10,000 s after H/D exchange of DJ-1 with or without peptide binding are shown. The deuteration differences are mapped onto the DJ-1 structure.

Reduction with 1 M DTT led to partial recovery of the activity of oxidized DJ-1; however, its activity remained lower than that of the originally reduced form. Therefore, we compared the structural differences of these forms through HDXMS. The results revealed that the DTT-reduced DJ-1 protein exhibited a significant increase in deuterium exchange at both the dimeric binding interface and the active catalytic triad, resulting in an overall increase in the deuterium exchange rate. The percentage difference for each fragment was computed by subtracting the H/D exchange percentage of DJ-1 before DTT reduction from that after DTT reduction, and these results are mapped onto the crystal structure in Figure 10A. After DTT reduction, increased deuterium exchange was observed in DJ-1, specifically in peptides 17–25 (17.6%), 17–26 (17.6%), 18–26 (16.9%), and 19–26 (23.3%) in the αA helix. An increase in deuterium exchange was also discovered in the central β2 sheet at the dimeric interface, with sequences 27–38 exhibiting a 27.3% increase and 31–38 exhibiting a 16.1% increase. Peptides related to the catalytic region—such as sequences 18–26 (16.9%) and 119–130 (6.2%)—as well as those near Cys106—including sequences 94–106 (6.1%), 104–112 (20.7%), and 105–112 (14.3%)—exhibited varying degrees of increased deuterium exchange. In the β6 sheet, peptides 69–82 exhibited an 18.2% increase, and peptides 69–84 exhibited a 19.5% increase. Furthermore, peptides 138–154 in sheets β9, β10, and β11 exhibited a 10.1% increase, and peptides 154–163 exhibited a 14.7% increase. Finally, in the hydrophobic core region, peptides 167–181 in the αG and αH helices exhibited a 5.8% increase, and peptides 179–195 exhibited a 6.6% increase. Overall, the H/D exchange of DJ-1 after DTT reduction was significantly higher than that of unreduced DJ-1. To confirm the effectiveness of DTT reduction, we conducted the reduction reaction to the protein samples, which were stored apart in the freezer for four months, at two separate times (Supplemental Figure S5). The HDXMS results at 10,000 s showed no difference.

An analysis of the variation in H/D exchange through the addition of P1 and P2 to the originally reduced DJ-1 protein revealed greater differences compared with the results for the DTT treated protein (Supplemental Figure S6). This enabled the analysis of activation disparities between P1 and P2 (Figure 10B,C). Although both P1 and P2 perturb DJ-1 dimerization, these two peptides yielded distinct levels of perturbation. The overall results are consistent with the results obtained through HDXMS for the DTT-reduced protein, but more prominent differences were found for P1 and P2 treatments. Both P1 and P2 resulted in reduced H/D exchange at the dimerization interface and increased H/D exchange surrounding the active site. By contrast, at the central β sheets, P1 addition led to a decrease in deuteration on the β1 and β6 sheets, whereas P2 addition enhanced deuteration in the same region. We further confirmed that DTT treatment did not have any structural effects on the originally reduced DJ-1 by HDXMS (Supplemental Figure S7).

## 3. Discussion

### 3.1. DJ-1 Dimerization and Activity

DJ-1 activity is essential for maintaining mitochondrial health during periods of oxidative stress. Experimental findings and crystal structure analyses revealed the dimeric nature of DJ-1. After the deglycation activity of purified DJ-1 was confirmed, DSS cross-linking experiments were conducted to verify the dimeric conformation of DJ-1 (Figure 2C). The results of H/D exchange indicated relatively low deuterium exchange levels in the outer regions of the protein, specifically in the αA helix and β4 sheet. The X-ray crystal structure of DJ-1 in its dimeric form (PDB: 2OR3) revealed that the αA helix forms the core of the dimeric interface, whereas the β4 sheet is situated on the side of the dimeric binding interface. These regions precisely align with the dimeric binding interface of DJ-1, thereby reducing the solvent accessibility of the αA helix and β4 sheet. The L166P mutation destabilizes the αG-helix and disrupts the homodimer, resulting in activity loss. However, the impact of this point mutation on the overall structure of the DJ-1 monomer remains unclear, and whether the monomeric form of DJ-1 exhibits lower activity is yet to be confirmed. The αG-helix plays a critical role in the activity of DJ-1. Although the monomer form of DJ-1 could not be obtained for a direct HDXMS comparison with the dimeric form, our findings suggest that the fast-exchanging form with high solvent accessibility may have lower activity (Figure 10A). The DJ-1 monomer can be assumed to have higher solvent accessibility than the DJ-1 dimer.

Cys106, Glu18, and His126, situated on distinct loops, collectively form the catalytic triad of DJ-1, a characteristic feature shared with other enzymes with an α/β hydrolase domain. The active site of Cys106 is a crucial residue governing the deglycation activity of DJ-1, and the microenvironment surrounding this active site plays a crucial role in ensuring the optimal activity of DJ-1. The oxidation of Cys106 enhances deglycation activity, but excessive oxidation of Cys106 leads to loss of activity [35]. Further analysis of the dimeric form of DJ-1 revealed hydrogen bonding between Cys106 and Glu18. Any disruption in the dimerization can induce a change in the shape of the active site, thereby altering catalytic activity. Thus, alterations in the conformation around the active site can differentially influence the catalytic activity of DJ-1.

### 3.2. Dimeric DJ-1 Binding with P1 and P2

The dimeric configuration of DJ-1 was demonstrated to exhibit superior deglycosylation activity and neuroprotective effects compared with the monomeric form [41]. To modulate DJ-1’s deglycation activity, we designed two peptides targeting the dimeric structure of DJ-1. Initially, our hypothesis suggested that these peptides, upon binding to DJ-1, would disrupt the dimeric structure, thereby altering DJ-1’s activity. However, subsequent cross-linking reactions conducted using DSS and the analysis of SDS-PAGE results revealed that the DJ-1 remained in its dimeric form. Further investigation through HDXMS analysis enabled quantification of the impact of the designed peptides on the enzyme. Remarkably, the HDXMS results not only confirmed the binding of the peptides to the dimeric structure of DJ-1 but also revealed an unexpected outcome. Instead of destabilizing the DJ-1 dimer, these peptides had a stabilizing effect and enhanced the activity of DJ-1.

The HDXMS data of DJ-1 with P1 and P2 revealed substantial changes, confirming the binding of these peptides to DJ-1. If P1 and P2 could disrupt the dimeric structure of DJ-1, their addition should result in an overall increase in deuterium exchange, potentially leading to a decrease in deuteration near the αA helix (16E–28R) at the P1 binding site. The HDXMS results revealed a decrease in deuterium exchange at the αA helix positions (16E–28R), with decreases of 10.1% and 31.5% at peptides 17–25 and 19–26, respectively, at 10,000 s. However, at the binding interface on the opposite side of the dimer, near the β4 sheet (50V–53C), a clear difference was not found at peptides 37–51 and 46–57. Importantly, no increase in deuteration of the entire enzyme was observed, particularly around the active sites. This finding indicated that although P1 can interact with DJ-1, this interaction may not be sufficient to completely disrupt the dimeric structure of DJ-1. Similarly, in the P2 experiment, where P2 is expected to bind to the β4 sheet (V50–C53), the results revealed decreases in deuterium exchange at 10,000 s for sequences 37–51 (−19.8%), 48–54 (−6.4%), and 50–58 (−10.1%). However, at the αA helix (16E–28R) positions, no increase was found; instead, a decrease was observed in the HDXMS analysis. This indicates that both P1 and P2 are unable to disrupt the dimeric structure of DJ-1. The validation of the DSS cross-linking reactions and the results of H/D exchange indicate that P1 and P2 do not disrupt the dimeric structure of DJ-1 and that they induce structural changes in the protein, enhancing its deglycation activity.

### 3.3. Change in Activity Mechanisms of DJ-1 after P1 and P2 Binding

The activity tests of DJ-1 revealed that both P1 and P2 effectively enhanced the deglycation activity of DJ-1. The addition of P1 resulted in a decrease in deuterium exchange at 10,000 s for peptides 27–38 (−23.3%) and 31–38 (−18.2%). These decreases may be attributable to the binding of P1 to the αA helix, which made the surrounding structure more compact and led to structural hindrance and a decrease in deuterium exchange in these two regions. Similarly, the addition of P2 led to a decrease in deuterium exchange at 10,000 s for peptides 37–51 (−19.8%) and 19–26 (−27.4%). This decrease may be attributable to the binding of P2 to the β4 sheet, which induced structural hindrance and reduced deuterium exchange in these sequences. Additionally, both P1 and P2 resulted in a decrease in deuterium exchange in peptides 11–17, 17–25, 119–130, and 129–139. These regions are associated with the catalytic triad structure containing Cys106, Glu18, and His126. Studies have indicated that the formation of bonds with SO_2_^−^ by E18, H126, and oxidized Cys106 after oxidation results in enhancement of the activity of DJ-1 [35]. The binding of SO_2_^−^ stabilized Cys106 in the active sites and facilitated hydrogen dissociation of the thiol on cysteine. This stabilized structure led to improved solvent accessibility and reactivity, enhancing substrate binding, and increasing the activity of DJ-1.

Although both P1 and P2 were found to effectively enhance the deglycation activity of DJ-1, they employ distinct mechanisms to regulate this activity. The HDXMS results indicated similar effects on the active sites, dimer-binding region, and hydrophobic core, suggesting that both peptides activate DJ-1 by binding to the dimer-binding regions, inducing crowding effects in the dimer and hydrophobic core regions. This, in turn, stabilizes Glu18, His126, and Cys106, increasing substrate affinity and enhancing the deglycation activity of DJ-1. However, on the β sheet core of DJ-1, P1 tightens the structure of the β1 and β6 sheets, whereas P2 exerts a loosening effect on these sheets. This discrepancy may be attributable to the distinct binding regions of P1 and P2. When P1 binds to the dimer-binding interface at the αA helix through hydrophobic interaction, crowding effects are created in the central structure of the dimer-binding interface and exert an inward force that compresses the adjacent β1 and β6 sheet structures. By contrast, P2, located on the side of the dimer-binding interface, exerts binding interactions that function as a pulling force toward the α/β hydrolase core. This simultaneous action stretches the structures of the β1 and β6 sheets outward, resulting in loosening of the structure.

### 3.4. Oxidized DJ-1 Reduced by DTT

After the protein was purified, its SDS-PAGE pattern contained two distinct bands, with the upper band indicating significantly higher deglycation activity than that of the two-band DJ-1. Notably, the lower band of the protein could be reduced to a single band of DJ-1 through the addition of a high concentration of DTT, resulting in increased activity. However, despite the SDS-PAGE patterns of both the original DJ-1 and the DTT-reduced DJ-1 containing a single band, differences in activity persisted, as evidenced by the H/D exchange data. In the DTT-reduced DJ-1, the overall protein structure became more relaxed, including the αA helix; the β2 and β4 sheets in the dimeric functional region; the catalytic triad (C106, E18, H126) in the active catalytic region; the αC, αD, and αE helices; the β6, β8, β9, β10, and β11 sheets in the protein backbone region; and the αG and αH helices in the hydrophobic core region. Deuterium exchange increased in all these regions. However, a decrease in deuterium exchange was discovered in specific regions, such as the αD helix in the protein backbone (sequences 78–91, −6.2%), the β1 sheet (sequences 2–10, −7.1%), and a loop structure in the αE helix to the middle of the β8 sheet (sequences 107–120, −47.9%). The H/D exchange data revealed that although DTT can reduce the amount of oxygen in DJ-1, oxidation may induce irreversible changes in the structure of DJ-1. In particular, in the catalytic mechanism, DJ-1 is hypothesized to adjust its structure for activation by contracting the dimeric and catalytic functional regions. However, in the DTT-reduced DJ-1, these functional regions were in a more relaxed state, indicating a strong correlation between the activity regulation of DJ-1 and these two functional regions.

In the DTT-reduced DJ-1 protein, the structure near the active site C106 was found to be relaxed. When C106 was oxidized, steric hindrance occurred and was speculated to create an outward pushing force and disrupt the catalytic triad structure. Removal of oxygen atoms from the oxidized Cys106 failed to restore the correct catalytic triad, leading to relaxation of structures near C106, such as the αC and αE helices, β6 and β11 sheets, and the surrounding loop structures. Simultaneously, the dimeric structure’s αA helix and β2 sheet also became relaxed, resulting in the overall alteration of the protein backbone structure.

### 3.5. DJ-1 Peptide Activation

Given the crucial role of dimerization in regulating DJ-1 activity, disrupting dimerization is a natural strategy for modulating DJ-1 activity. Two peptide sequences from the dimerization interface were designed as potential modulators. Notably, both peptides were found to enhance the deglycation activity of DJ-1 in vitro, but they exhibited different effects on the catalytic site.

P1, located in region 15–29 and containing the critical Glu18, competes with the dimerization interface and rearranges the geometry of the catalytic triad. HDXMS analysis revealed a significant reduction in deuteration at the direct binding region, which extends to the entire 11–38 region. Notably, the other dimerization region remains unaffected or, at least, does not loosen, corresponding to the maintained dimer structure. The catalytic triad forms a rigid structure with Glu18 and His126, but Cys106 seems to be in a more relaxed loop.

P2, located in the 47–56 region, forms a β-sheet in the homodimer structure, interacting antiparallelly with the same β-sheet of the dimer partner protein. Although the competition of P2 toward the dimerization interface does not break the dimer, it leads to a decrease in H/D exchange, indicating a more compact structure at the interface. Although P2 insertion may tighten the dimer and optimize the catalytic triad, the overall α/β hydrolase domain remains in a relaxed conformation.

## 4. Material and Methods

### 4.1. Materials

pET3a-His-DJ1 was purchased from Addgene, Watertown, MA, USA (plasmid number 51488), originally a gift from the Michael J Fox Foundation, New York City, NY, USA. Imidazole, tween 20, DTT were purchased from Amresco LLC, Solon, OH, USA. A 30% LB broth powder, lysozyme, and IPTG were purchased from Chumeia, Hsinchu City, Taiwan. β-Mercaptoethanol(β-ME) was purchased from Calbiochem, St. Louis, MO, USA. BCIP (5-bromo-4-chloro-3-indolyl phosphate) and guanidine hydrochloride (GuHCl) were purchased from Cyrusbioscience, New Taipei City, Taiwan. Glycerol, TEMED, and DJ-1 antibody were purchased from Invitrogen, Waltham, MA, USA. Triton, SDS, and ammonium persulfate (APS) were purchased from OmniPur, Radnor, PA, USA. Ni-NTA agarose was purchased from QIAGEN, Hilden, Germany. *N*-acetyl-l-cysteine, methylglyoxal solution (MGO), pepsin, NBT (Nitro blue tetrazolium chloride), phenylmethyl sulfonyl fluoride (PMSF), formic acid (FA), anti-rabbit IgG, and D2O were purchased from Sigma-Aldrich, St. Louis, MO, USA. Protein assay dye was purchased from Bio-Rad, Hercules, CA, USA. Immobilized pepsin and protein stain were purchased from Thermo Fisher Scientific, Waltham, MA, USA. Peptide 1 (^15^EEMETIIPVDVMRRA^29^) and peptide 2 (^47^SRDVVICPDA^56^) were synthesized by Yao-Hong Biotechnology, New Taipei City, Taiwan.

### 4.2. Protein Purification

pET3a-His-DJ1 transformed BL21 and was grown at 37 °C to OD = 0.45. Protein expression was induced by 0.1 mM IPTG at 30 °C for 2 h. The cell lysate was collected by 10,000 rpm, centrifuged at 4 °C for 30 min and stored at −80 °C. The cell lysate was defrosted for 10 min on ice before addition of 20 mL of lysis buffer, containing 50 mM Tris pH 8, 150 mM NaCl, 1 mg/mL lysozyme, 1 mM PMSF, 20 mM β-ME, and 0.1% Triton.

After being thoroughly vortexed, the cell lysate was homogenized at 65% 125 W sonication on ice (QSonica Q125, 6.4 mm probe, from QSonica LLC., Newtown, CT, USA). The sonication was in 10 cycles of 30 s sonication and 30 s stops. After 10,000 rpm centrifugation for 30 min, the collected supernatant was flowed through 2 mL Ni-NTA column. The Ni-NTA column was pre-washed with 2 mL of washing buffer (50 mM Tris pH 8, 150 mM NaCl, 20 mM β-ME, 10 mM Imidazole). The column with bound protein was washed with 40 mL of washing buffer. After washing, the protein was eluted by elution buffer (50 mM Tris pH 7.5, 125 mM NaCl, 3 mM DTT, 150 mM Imidazole, 50% Glycerol). The eluate fractions were collected every 0.5 mL. The protein concentrations were determined by Bradford assay (Bio-RAD Laboratories, Hercules, CA, USA) and quantitated by a 96-well plate reader at 595 nm absorbance. Protein was verified by Western blotting and the purity were estimated by SDS-PAGE.

### 4.3. DJ-1 Activity Assay

To measure the activity of DJ-1, the fresh substrate was prepared immediately before the assay. PBS buffer was bubbled by nitrogen gas for 3 min before experiments. The glycation reactions were initiated by mixing 0.5 mL of PBS buffer, 5 µL of 1M *N*-acetyl-L-cysteine (10 mM final concentration), and 7.82 µL of 0.65 M methylglyoxal solution (8 mM final concentration). After mixing, the sample was purged by nitrogen gas for 15 s and sealed by parafilm. The samples were incubated in 37 °C water baths for 10 min. After adding 23 µL (1.266 µg/µL) of DJ-1, the samples were further incubated for 30 min, and then the absorbance at 288 nm was detected.

### 4.4. Electrophoresis of DJ-1 Dimer

Twelve milligrams of DJ-1 protein was treated by 25 mM DSS crosslinker (Disuccinimidyl substrate) at 25 °C for 30 min. Fifteen milligrams of 15-mer peptide 1 (P1) and 10 μg of 10-mer peptide 2 (P2) were added to interact with DJ-1. DSS-treated and peptide-added protein samples were loaded onto a 12% polyacrylamide gel for SDS-PAGE. For the native-PAGE, protein samples were loaded onto a 12% polyacrylamide gel without SDS. NativePAGE cathode buffer additive (20×) was added to the NativePAGE running buffer to prepare the buffer for the cathode chamber. The running buffer was used without additive for the anode.

### 4.5. Sequence Identification of the DJ-1 Proteolytic Fragment

Before analyzing the HDXMS data, the sequences of the detected pepsin-digested fragments were first identified by MS/MS. To prepare the pepsin column, a 2.1 mm × 35 mm empty column was packed by Thermo Scientific Pierce immobilized pepsin agarose, (Thermo Scientific, Waltham, MA, USA). The column was flushed with 0.1% formic acid at a flow rate of 0.1 mL/min. The above two steps were repeated 3 to 4 times until the pepsin column was filled. After DJ-1 was proteolyzed into fragments by pepsin, the fragments were collected by a micro RP peptide trap (OPTI-TRAPTM from optimize technology, Oregon City, OR, USA) and then separated by a reverse phase HPLC in a C18 column (BioBasic 18 LC Columns, Thermo Scientific, Waltham, MA, USA). The mobile phase was the gradient between Buffer A: 0.1% formic acid and Buffer B: 80% acetonitrile/0.02% formic acid. The flow rate was set to 0.15 mL/min and most peptides were eluted in 30 min. The peptides were fragmented by tandem mass spectrometry (MS/MS), and the MS/MS data were imported into the X! Tandem-parser-1.7.7 software to calculate the sequences of the fragments.

### 4.6. Sequence Identification by X! Tandem

DJ-1 MS/MS data were exported from Bruker DataAnalysis and the intensity threshold was set at 700. The MS/MS data and the sequence were imported to X! Tandem for sequence identification. The mass error was set at 500 ppm. The sequence of each fragment was further manually verified based on the primary mass and the matched product ions under X! tandem-parser-1.7.7.

### 4.7. Hydrogen/Deuterium Exchange Experiments

A 20× Tris buffer (1 M Tris-base, 2.5 M NaCl, pH 7.5) was diluted by 99.9% D_2_O to prepare a 1× D_2_O buffer. Then, 15 µL (50 µg) of DJ-1 protein and D_2_O buffer was incubated in a 37 °C water bath for 30 min. H/D exchange was initiated by mixing 15 µL DJ-1 protein with 45 µL D_2_O buffer in a 37 °C water bath for 10, 30, 100, 300, 1000, 3000, and 10,000 s. The reactions were quenched by adding 140 µL of quench buffer (0.5% formic acid, 1 M GuHCl).

### 4.8. Mass Spectrometry Analysis

The pepsin column connected with the peptide trap was precooled on ice and the pepsin was activated by flushing the column with 0.1% formic acid at 0.1 mL/min. The quenched deuterated samples were withdrawn by syringe and injected into the pepsin column. The column was washed with 0.1% formic acid at 0.5 mL/min in HPLC for 1.5 min. The pepsin column was then disconnected, and the peptide trap was reconnected to a C18 column. The peptides were then separated by HPLC and analyzed by mass spectrometer. The HPLC mobile phase was the gradient between Buffer A: 0.1% formic acid and Buffer B: 80% acetonitrile/0.02% formic acid. The flow rate was set to 0.15 mL/min and most peptides were eluted in 30 min. The C18 column, LC running buffer, and the tubing were covered by ice. The peptides of the protein were detected on the positive mode of the mass spectrometry and the mass detection range was 400 to 2000 *m*/*z*. The data were processed on Bruker DataAnalysis (ver.4.1).

### 4.9. DJ-1 HDXMS Calculation

The sequence, retention time, and charge of the identified peptide fragments were uploaded to the H/D exchange software HDExaminer 1.2 as the peptide pool. The non-deuterated MS data of DJ-1 were further imported to identify the peptic peak. The peptides with low intensity were excluded for further calculation, and this step served as a second filter for peptide identification. The masses of the fragment of the non-deuterated DJ-1 were the standards for all other H/D exchange samples. Then, the MS data of the H/D exchange samples at seven time points were imported into the HDExaminer. The average mass of each deuterated peptide that appeared at each time point was calculated. The mass shift at a specific time point after H/D exchange was the amount of deuteration.

## 5. Conclusions

Two peptides have been identified as enhancers of DJ-1 activity. To understand the mechanisms behind this enhancement, we employed hydrogen/deuterium exchange mass spectrometry. The findings revealed distinct binding mechanisms for each peptide with the DJ-1 protein, ultimately contributing to the observed increase in activity.

## Data Availability

Data is contained within the article and Supplementary Materials.

## Supplementary Materials

The following supporting information can be downloaded at https://www.mdpi.com/article/10.3390/ijms252011075/s1.
