# Supplementary material for: Peptide Activator Stabilizes DJ-1 Structure and Enhances Its Activity
Source: Int J Mol Sci. 2024 Oct 15;25(20):11075. doi: 10.3390/ijms252011075 (PMC11508141; doi:10.3390/ijms252011075)
Supplement: Supplementary file 1 [file ijms-25-11075-s001.zip › ijms-3235386-supplementary.pdf]

Supplemental Table S1. Identified peptides from pepsin-digested DJ-1

Start	End	Sequence
2	10	ASKRALVIL
9	16	ILAKGAEE
11	17	AKGAEEM
17	25	METVIPVDV
17	26	METVIPVDVM
18	24	ETVIPVD
18	26	ETVIPVDVM
19	26	TVIPVDVM
25	32	MRRAGIK
27	38	RRAGIKVTVAGL
31	38	LNGKEVAA
37	51	GLAGKDPVQCSRDVV
38	46	LAGKDPVQC
39	45	AGKDPVQ
46	57	CSRDVVICPDAS
48	54	RDVVICP
50	58	VVICPDASL
50	59	VVICPDASLE
50	62	VVICPDASLEDAK
57	67	SLEDAKKEGPY
59	69	EDAKKEGPYDV
65	78	GPYDVVVLPGGNLG
68	76	DVVVLPGGN
69	82	VVVLPGGNLGAQNL
69	84	VVVLPGGNLGAQNLSE
73	82	PGGNLGAQNL
76	83	NLGAQNLS
78	91	GAQNLSESAAVKEI
89	99	KEILKEQENRK
92	96	LKEQE
94	106	EQENRKGLIAAIC
102	119	IAAICAGPTALLAHEIGF
104	112	AICAGPTAL
105	112	ICAGPTAL
107	120	AGPTALLAHEIGFG
114	121	AHEIGFGS
119	130	FGSKVTTHPLAK
123	133	VTTHPLAKDKM
125	132	THPLAKDK

129	139	AKDKMMNGGHY
138	154	HYTYSENRVEKDGLILT
147	158	EKDGLILTSRGP
152	163	ILTSRGPGTSFE
153	166	LTSRGPGTSFEFAL
154	163	TSRGPGTSFE
158	166	PGTSFEFAL
167	181	AIVEALNGKEVAAQV
171	178	ALNGKEVA
179	185	AQVKAPL
179	195	AQVKAPLVLKDHHHHHH

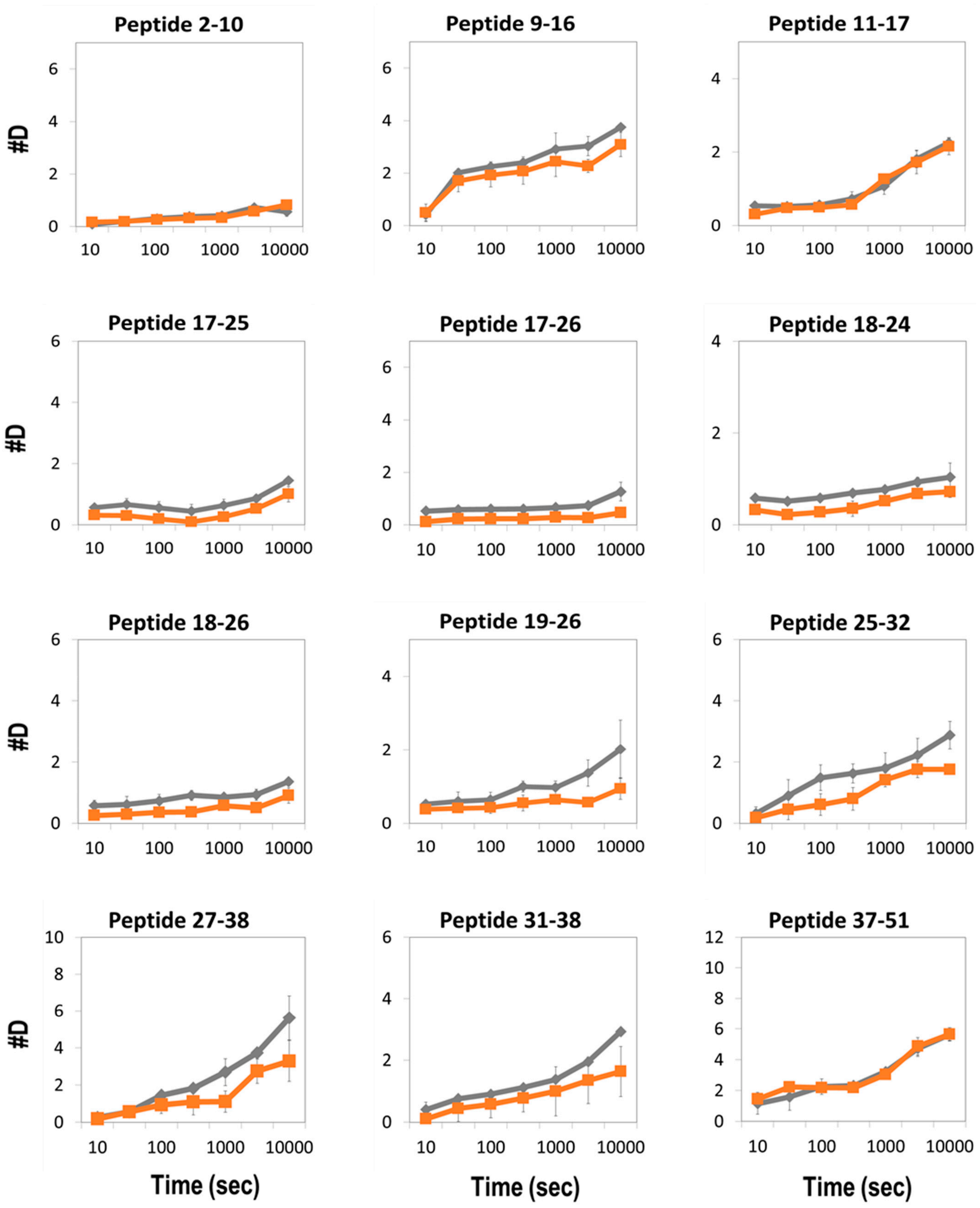

Peptide 2-10
Peptide 9-16
Peptide 11-17
Peptide 17-25
Peptide 17-26
Peptide 18-24
Peptide 18-26
Peptide 19-26
Peptide 25-32
Peptide 27-38
Peptide 31-38
Peptide 37-51
#D
Time (sec)

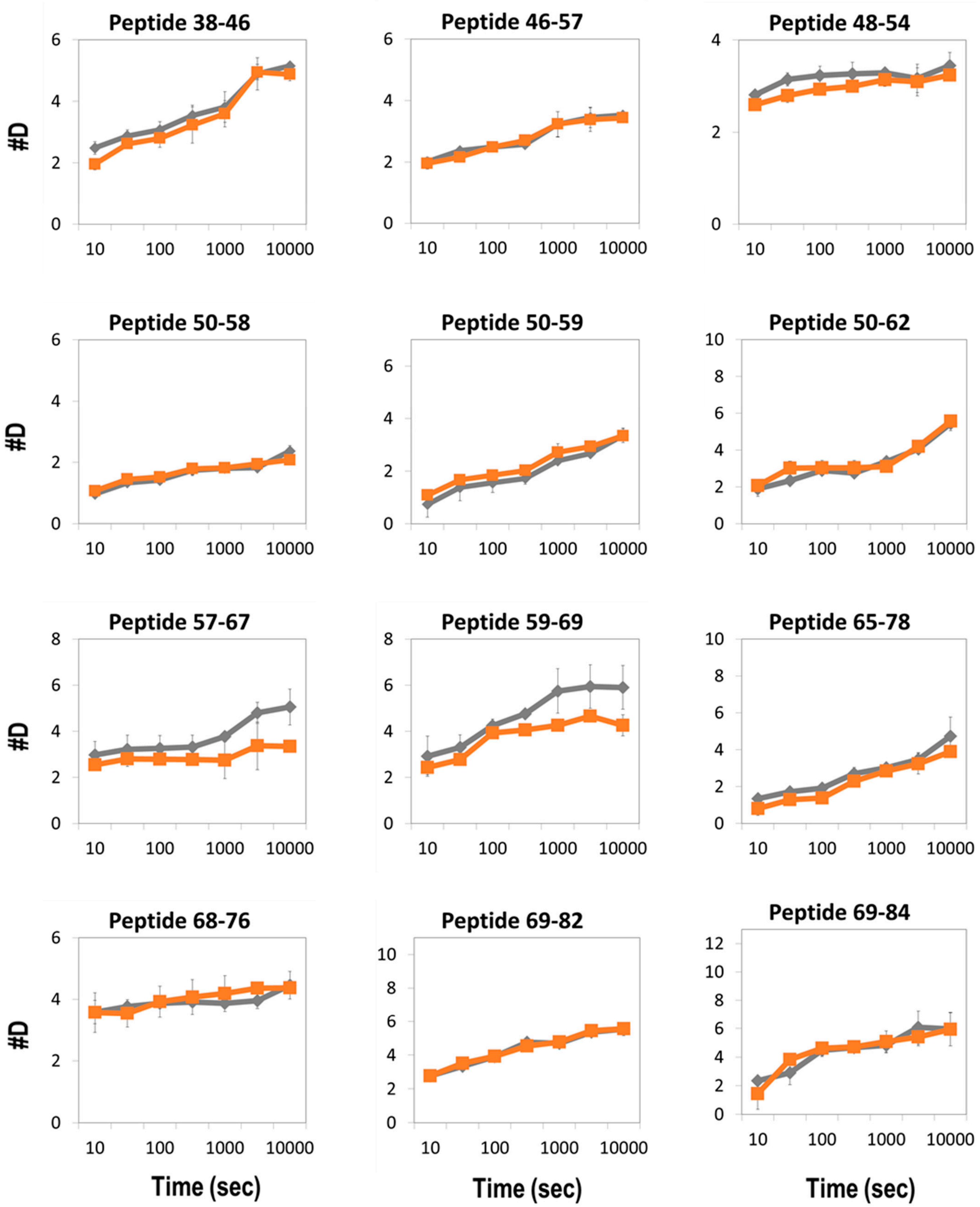
Peptide 38-46
Peptide 46-57
Peptide 48-54
Peptide 50-58
Peptide 50-59
Peptide 50-62
Peptide 57-67
Peptide 59-69
Peptide 65-78
Peptide 68-76
Peptide 69-82
Peptide 69-84
#D
Time (sec)

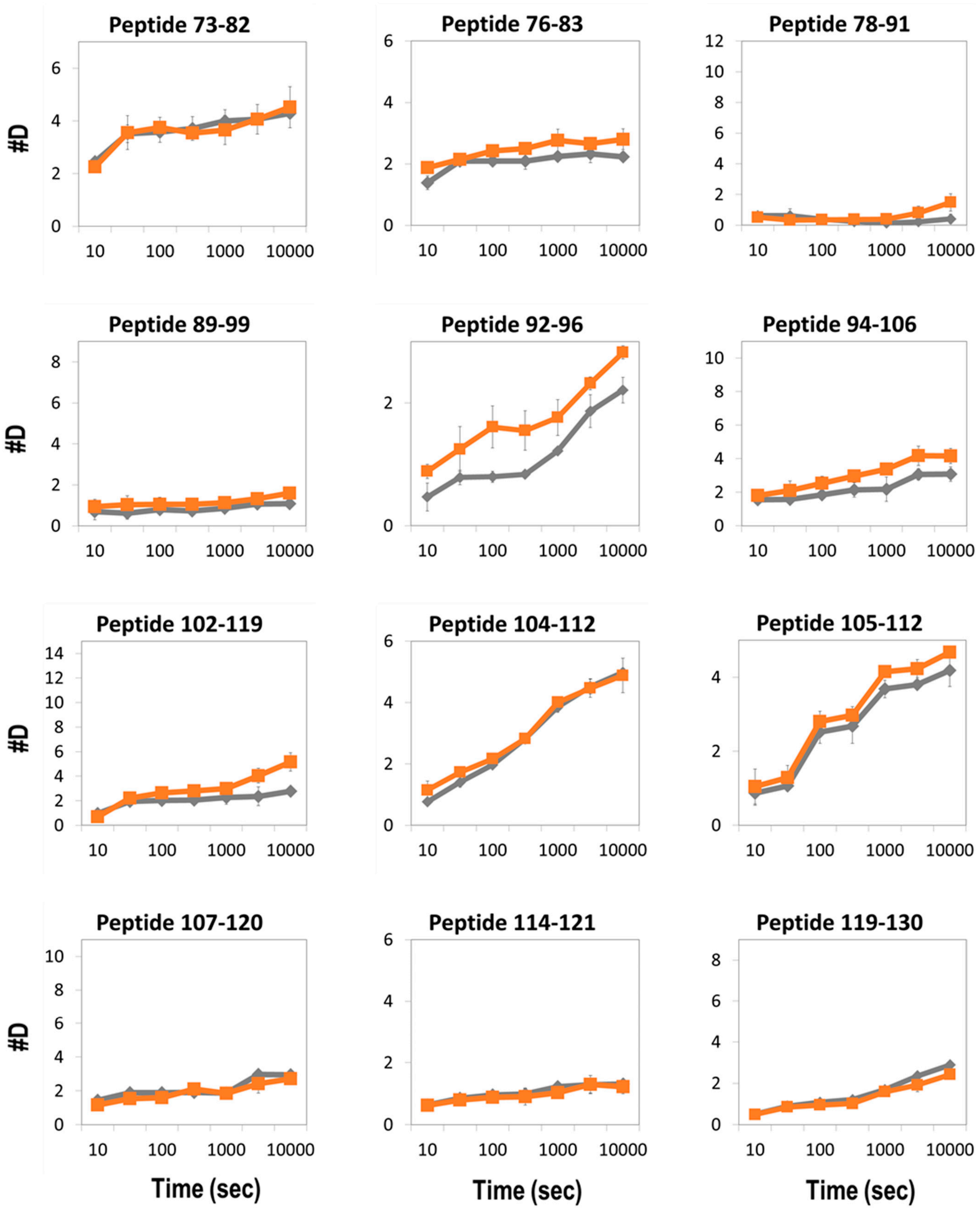
Peptide 73-82
Peptide 76-83
Peptide 78-91
Peptide 89-99
Peptide 92-96
Peptide 94-106
Peptide 102-119
Peptide 104-112
Peptide 105-112
Peptide 107-120
Peptide 114-121
Peptide 119-130
#D
Time (sec)
10
100
1000
10000

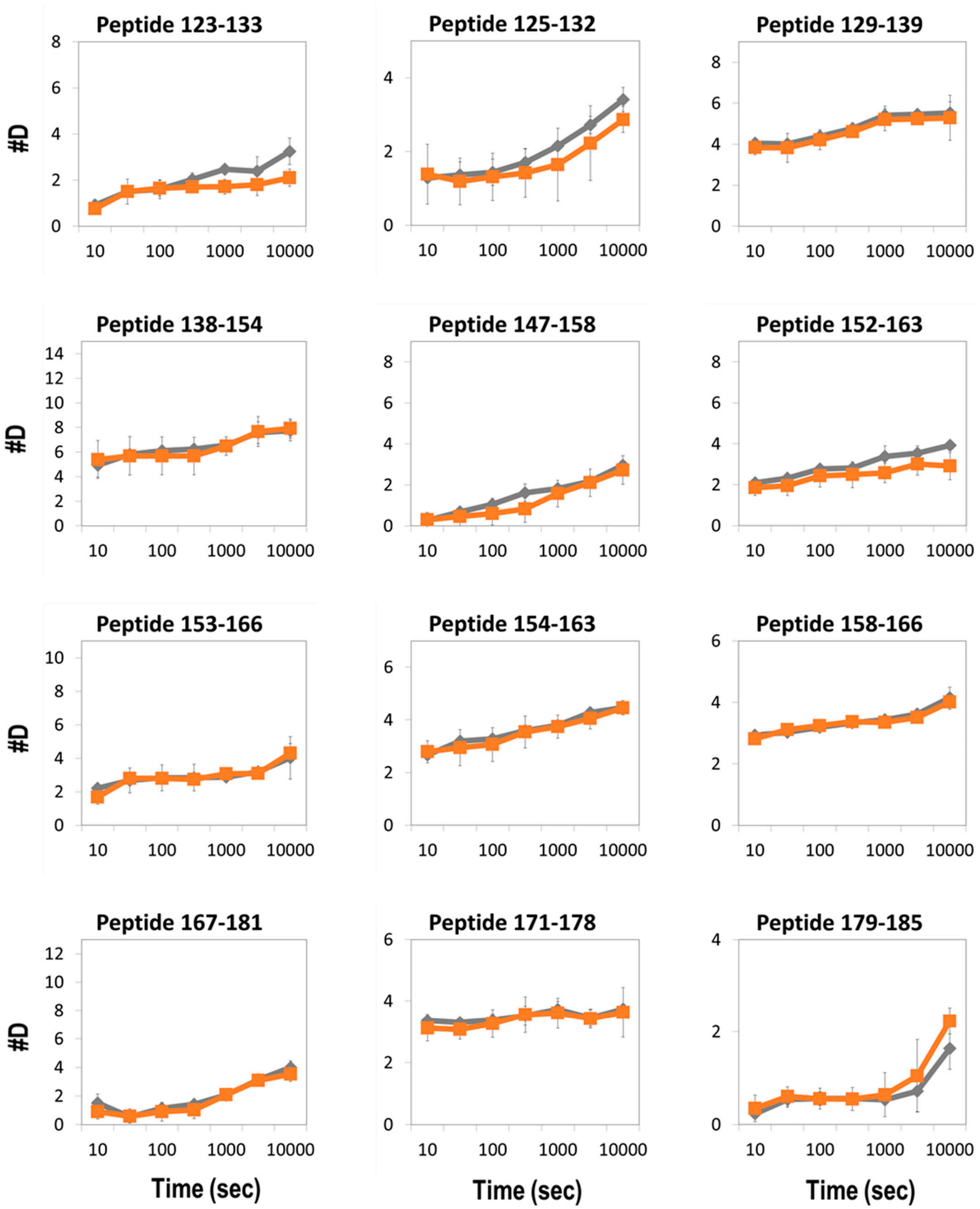
Peptide 123-133
Peptide 125-132
Peptide 129-139
Peptide 138-154
Peptide 147-158
Peptide 152-163
Peptide 153-166
Peptide 154-163
Peptide 158-166
Peptide 167-181
Peptide 171-178
Peptide 179-185
#D
Time (sec)
10
100
1000
10000

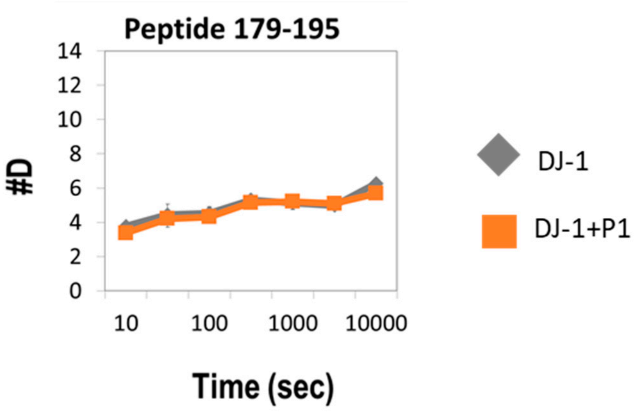

Supplemental Figure S1. Deuteration of DJ-1 and peptide 1 bound DJ-1 after H/D exchange.
DJ-1 bound to P1 was deuterated for 10, 30, 100, 300, 1000, 3000, and 10 000 s. The maximum value of the Y-axis is the maximum deuteration number of the peptides. All experiments were conducted in triplicate, and the errors represent the standard deviation.

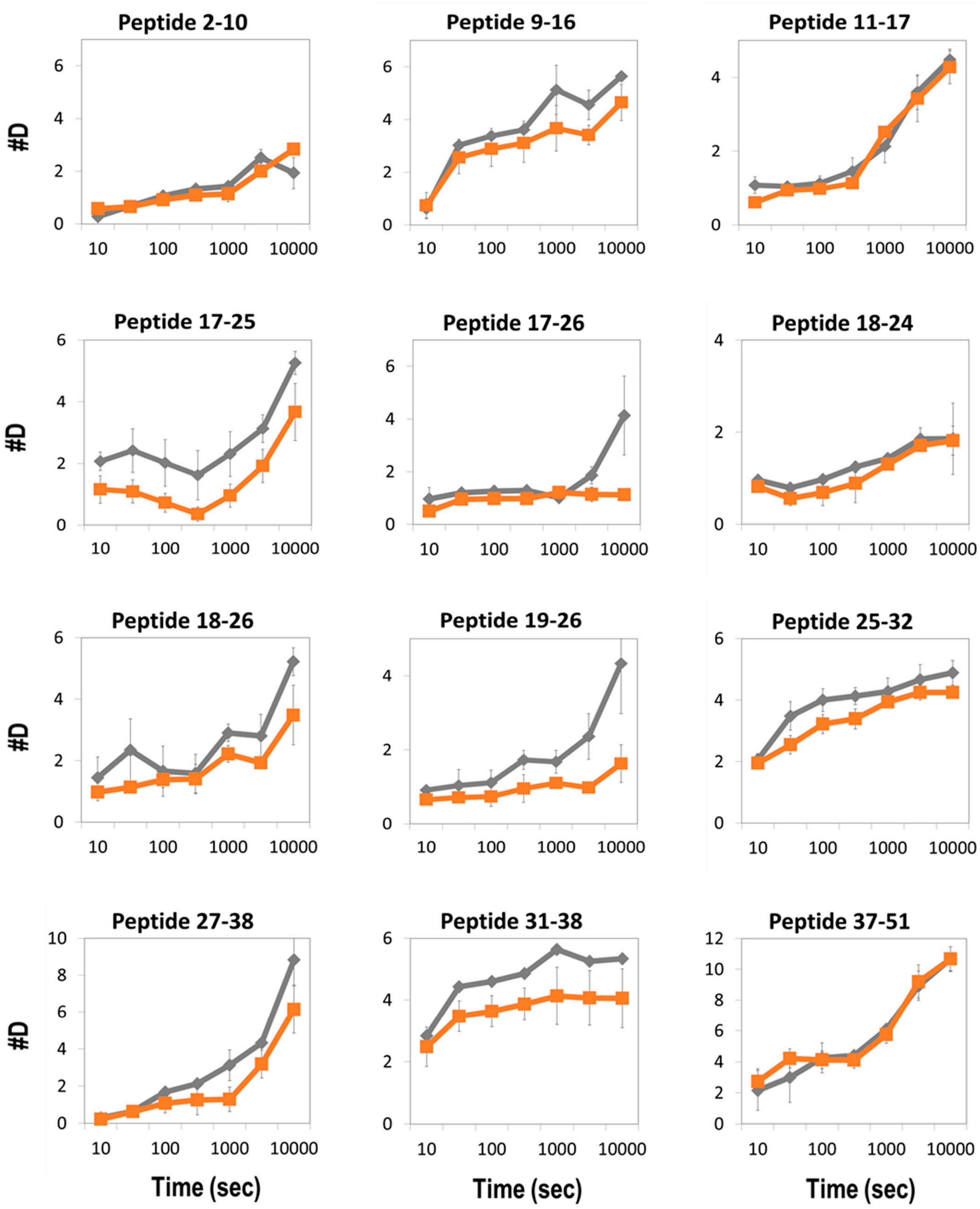

Peptide 2-10
Peptide 9-16
Peptide 11-17
Peptide 17-25
Peptide 17-26
Peptide 18-24
Peptide 18-26
Peptide 19-26
Peptide 25-32
Peptide 27-38
Peptide 31-38
Peptide 37-51
#D
Time (sec)

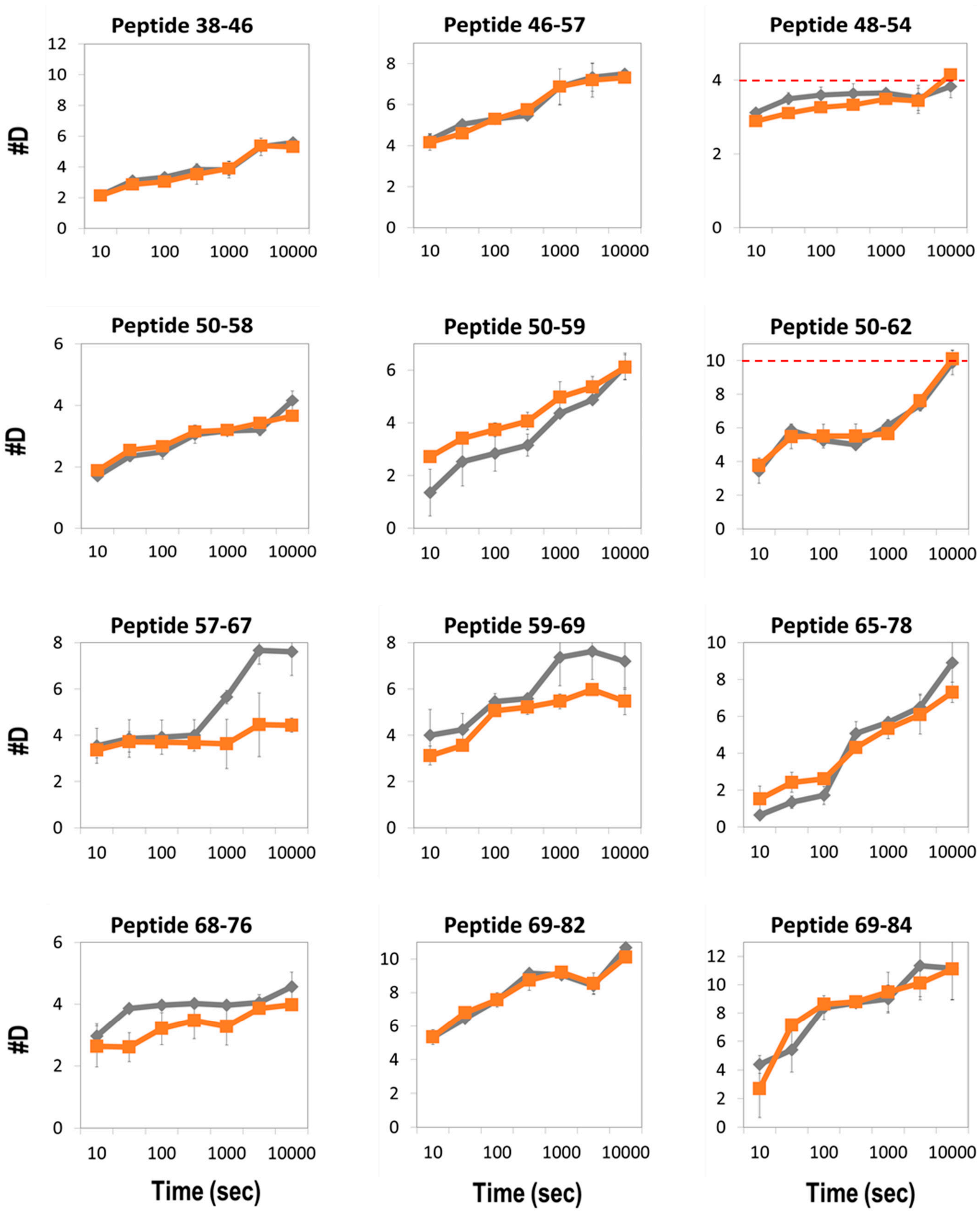
Peptide 38-46
Peptide 46-57
Peptide 48-54
Peptide 50-58
Peptide 50-59
Peptide 50-62
Peptide 57-67
Peptide 59-69
Peptide 65-78
Peptide 68-76
Peptide 69-82
Peptide 69-84
#D
Time (sec)

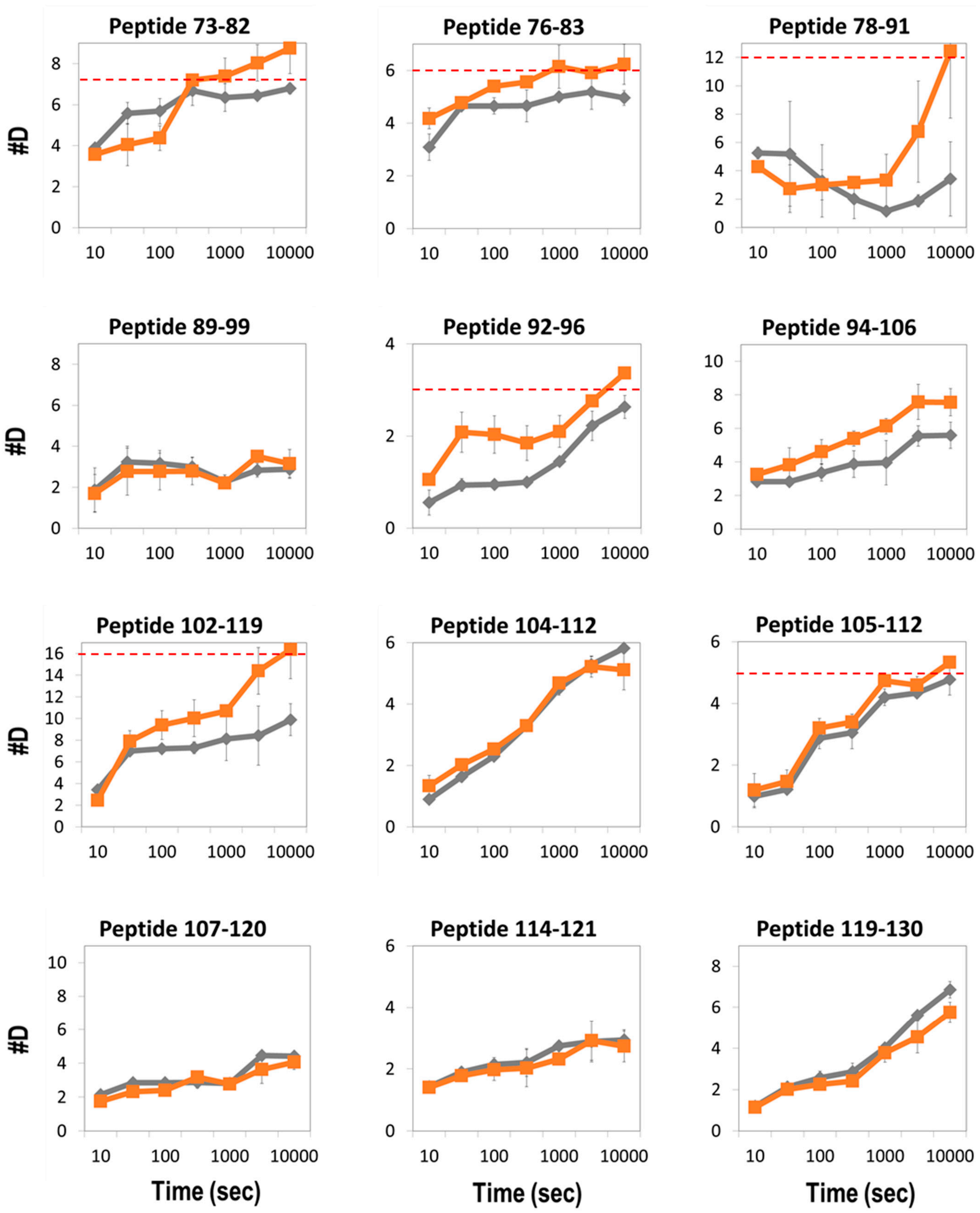

Peptide 73-82
Peptide 76-83
Peptide 78-91
Peptide 89-99
Peptide 92-96
Peptide 94-106
Peptide 102-119
Peptide 104-112
Peptide 105-112
Peptide 107-120
Peptide 114-121
Peptide 119-130
#D
Time (sec)
10
100
1000
10000

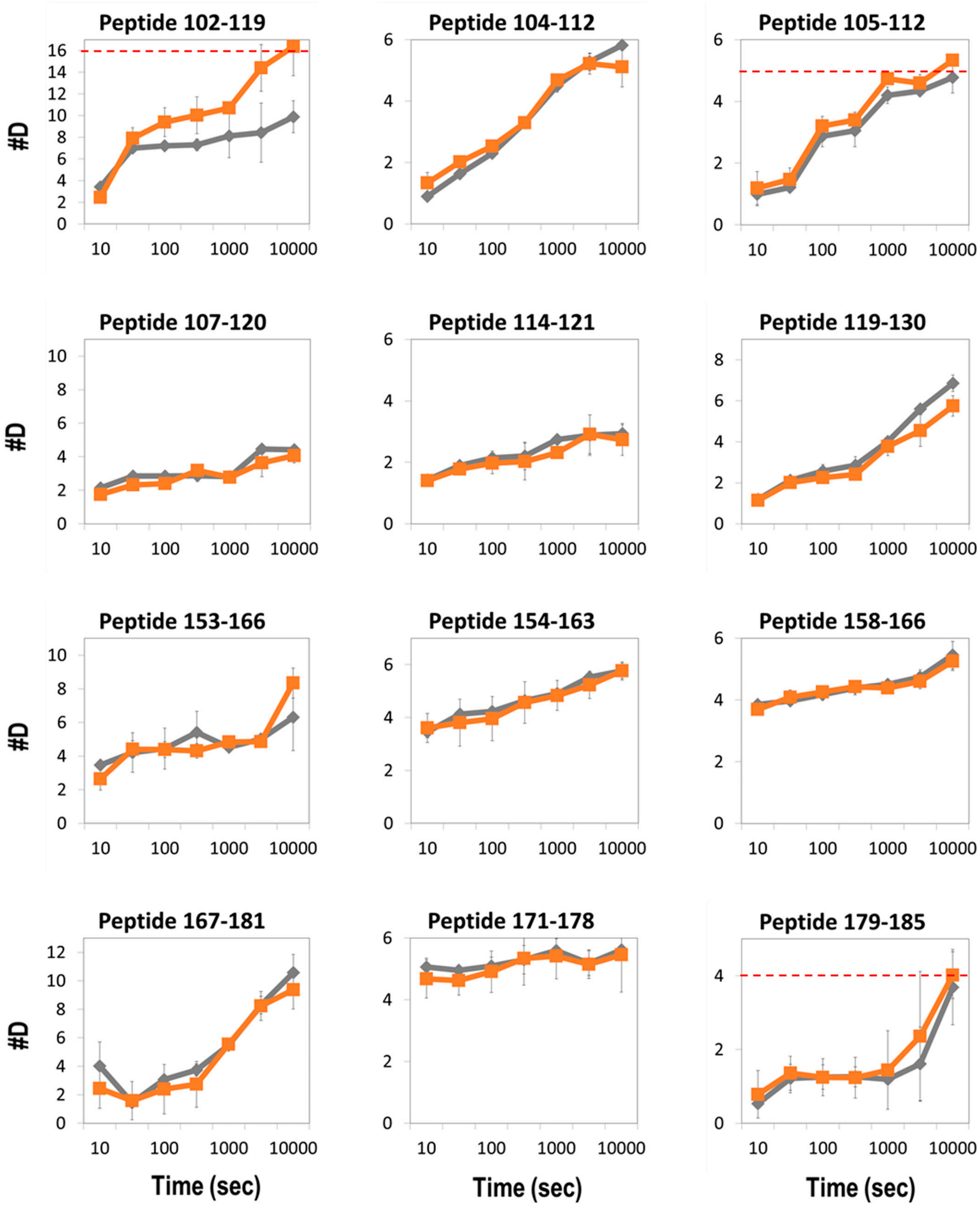

Peptide 102-119
Peptide 104-112
Peptide 105-112
Peptide 107-120
Peptide 114-121
Peptide 119-130
Peptide 153-166
Peptide 154-163
Peptide 158-166
Peptide 167-181
Peptide 171-178
Peptide 179-185
#D
Time (sec)

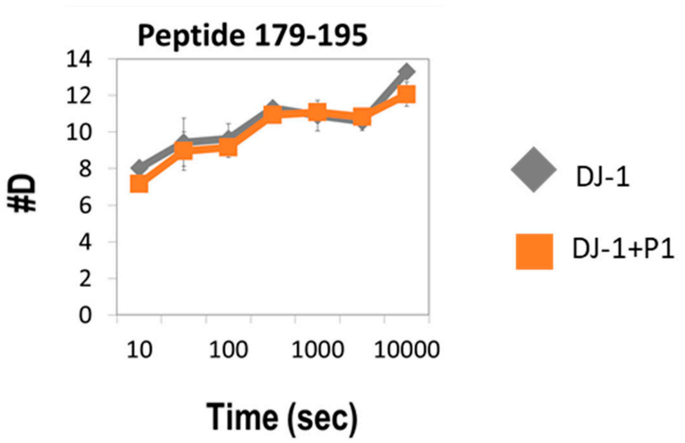

Supplemental Figure S2. H/D exchange results of DJ-1 and peptide 1 bound DJ-1 after back-exchange correction.

DJ-1 bound to P1 was deuterated for 10, 30, 100, 300, 1000, 3000, and 10 000 s. The number of deuteration were adjusted based on the 24-hour back exchange. The maximum value of the Y-axis or at the red dash line is the maximum deuteration number of the peptides. All experiments were conducted in triplicate, and the errors represent the standard deviation.

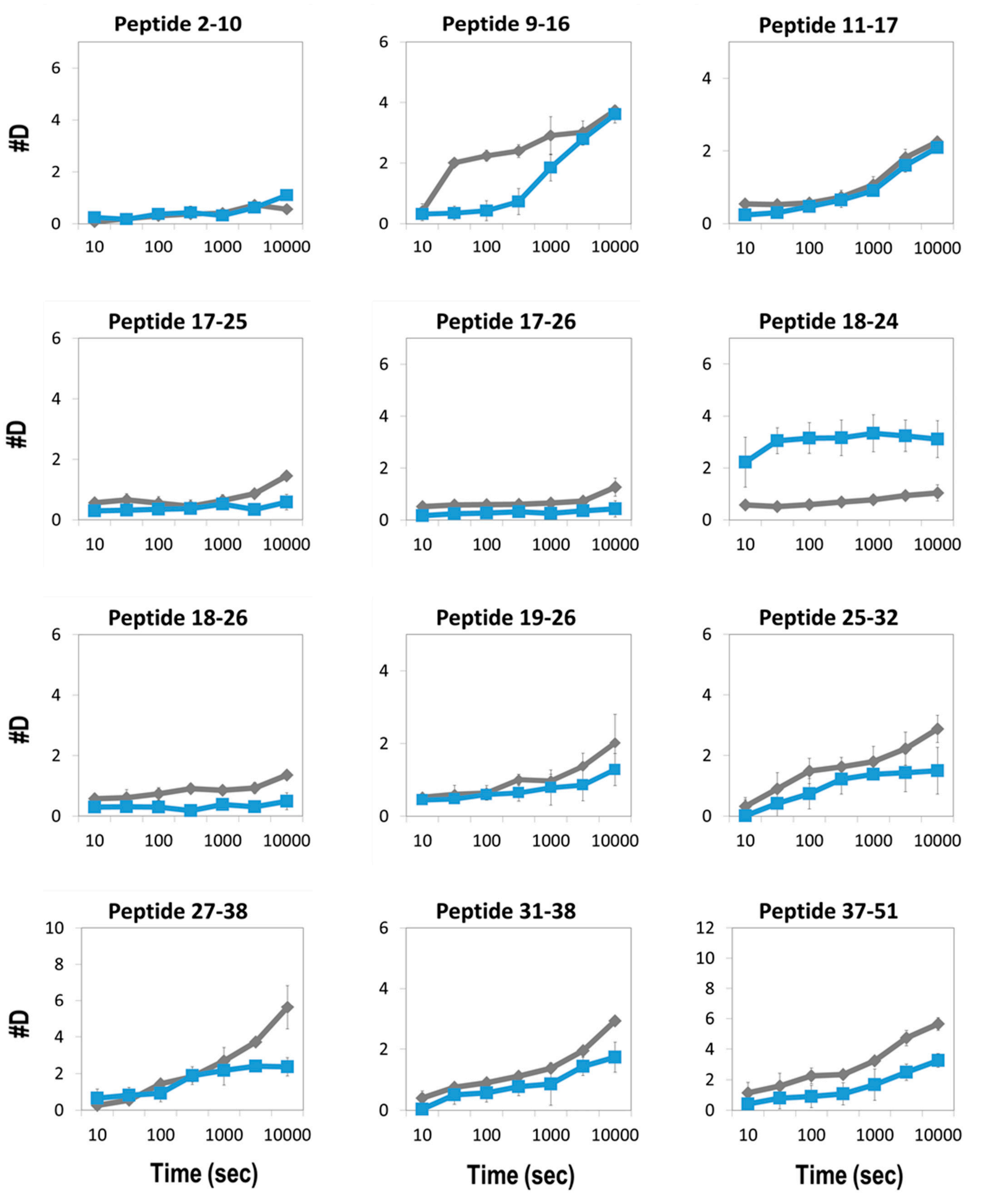
Peptide 2-10
Peptide 9-16
Peptide 11-17
Peptide 17-25
Peptide 17-26
Peptide 18-24
Peptide 18-26
Peptide 19-26
Peptide 25-32
Peptide 27-38
Peptide 31-38
Peptide 37-51
#D
Time (sec)
10
100
1000
10000

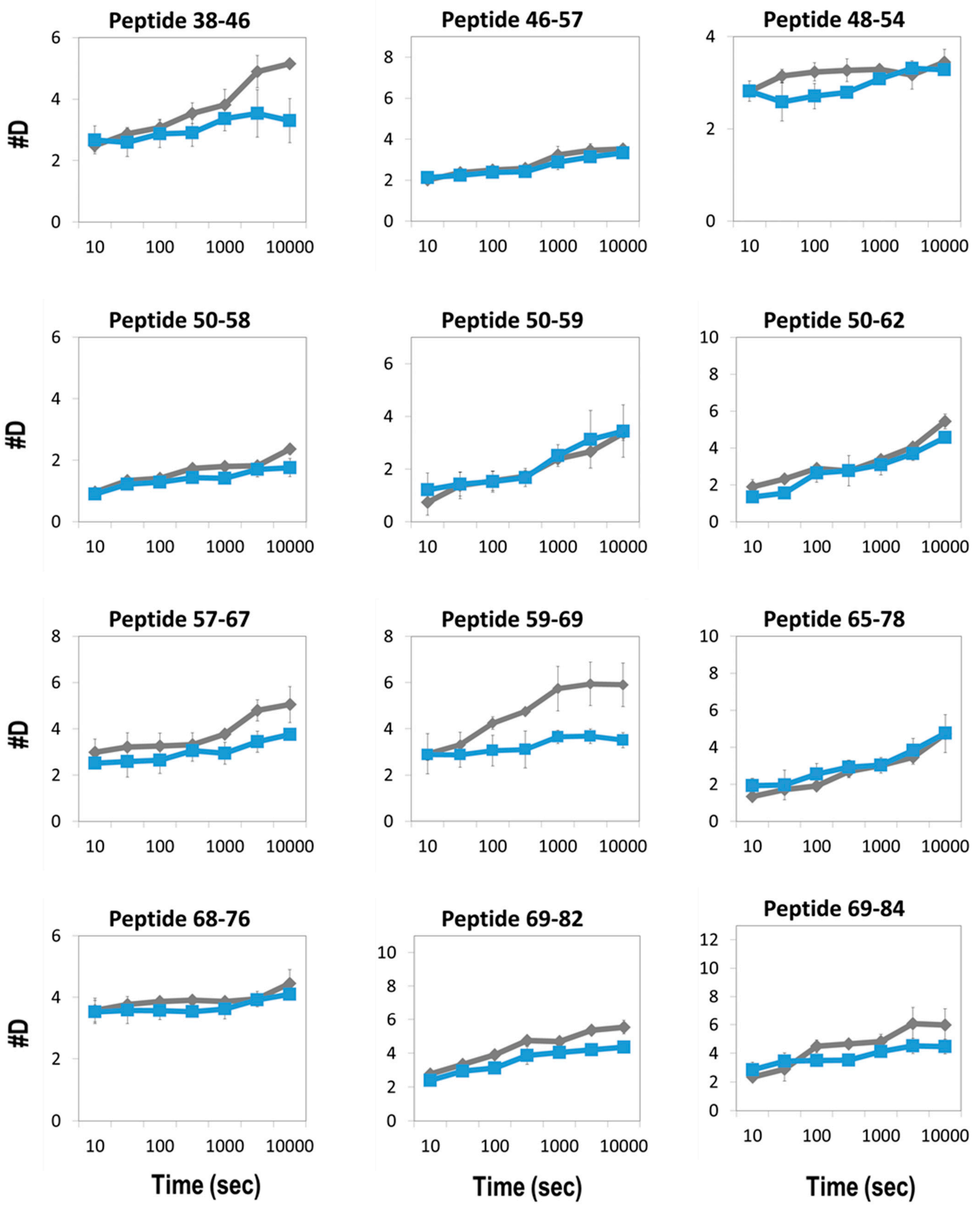
Peptide 38-46
Peptide 46-57
Peptide 48-54
Peptide 50-58
Peptide 50-59
Peptide 50-62
Peptide 57-67
Peptide 59-69
Peptide 65-78
Peptide 68-76
Peptide 69-82
Peptide 69-84
#D
Time (sec)

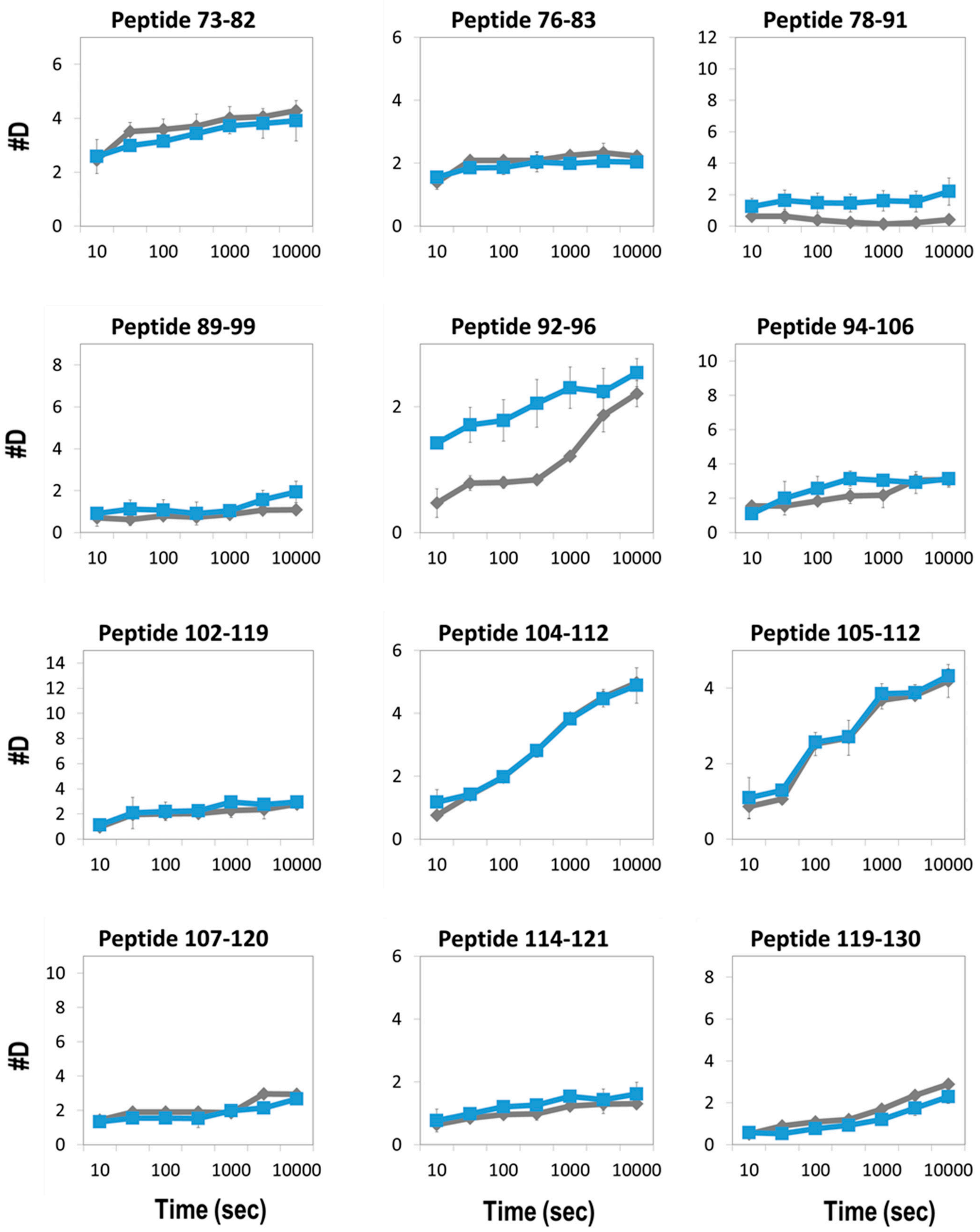
Peptide 73-82
Peptide 76-83
Peptide 78-91
Peptide 89-99
Peptide 92-96
Peptide 94-106
Peptide 102-119
Peptide 104-112
Peptide 105-112
Peptide 107-120
Peptide 114-121
Peptide 119-130
#D
Time (sec)

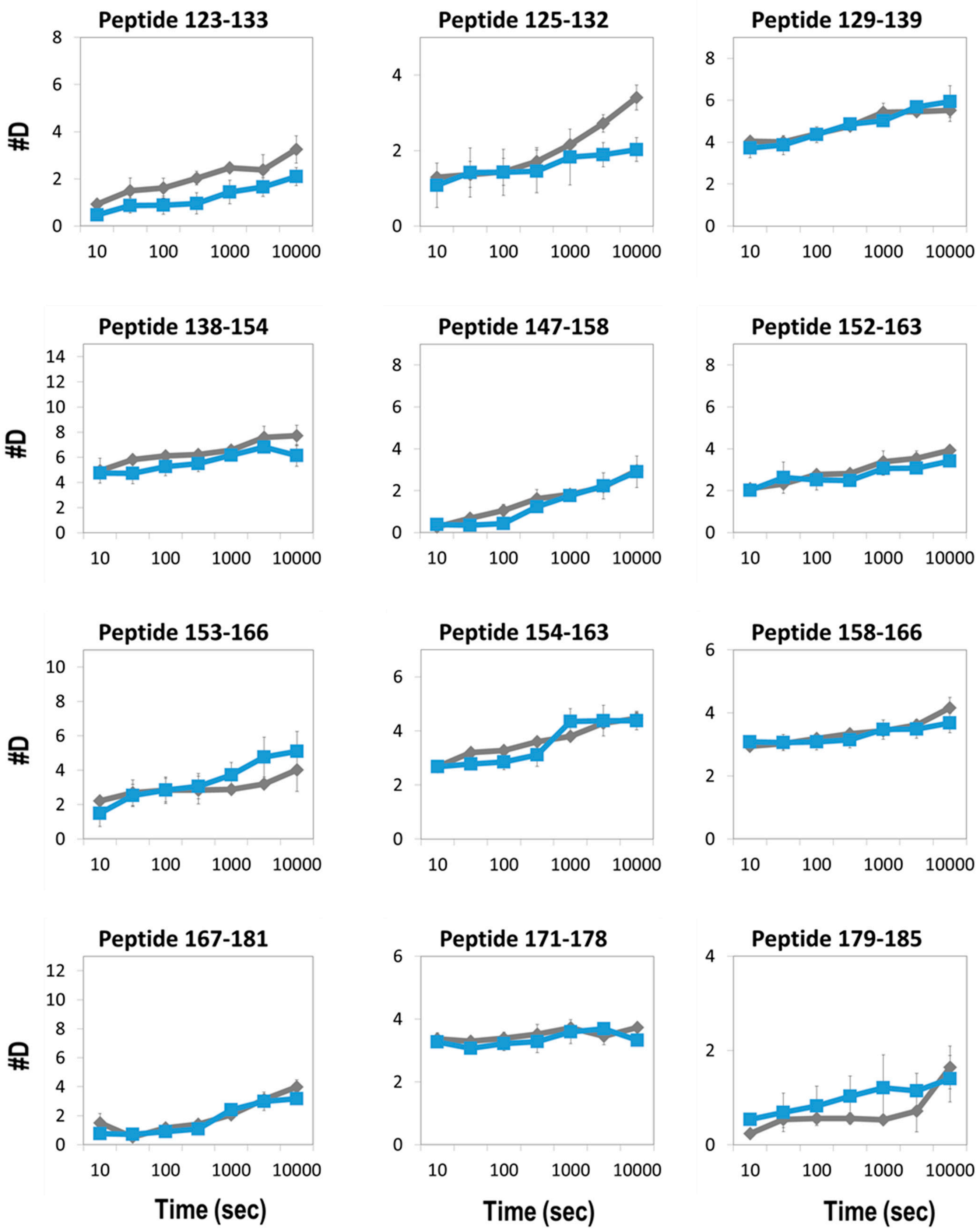

Peptide 123-133
Peptide 125-132
Peptide 129-139
Peptide 138-154
Peptide 147-158
Peptide 152-163
Peptide 153-166
Peptide 154-163
Peptide 158-166
Peptide 167-181
Peptide 171-178
Peptide 179-185
#D
Time (sec)
10
100
1000
10000

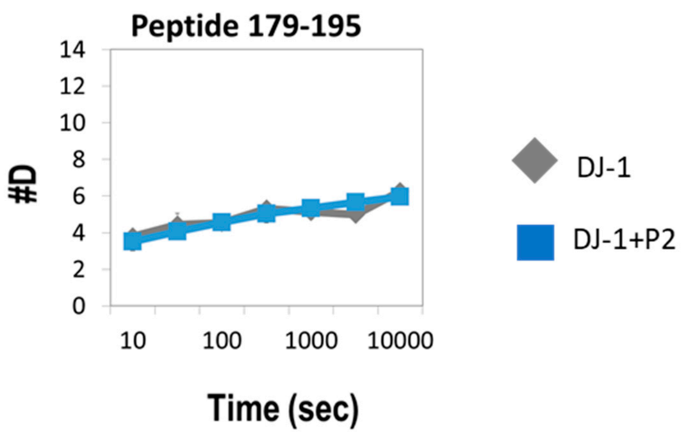

Supplemental Figure S3. Deuteration of DJ-1 and peptide 2 bound DJ-1 after H/D exchange.
DJ-1 bound to P2 was deuterated for 10, 30, 100, 300, 1000, 3000, and 10 000 s. The maximum value of the Y-axis is the maximum deuteration number of the peptides. All experiments were conducted in triplicate, and the errors represent the standard deviation.

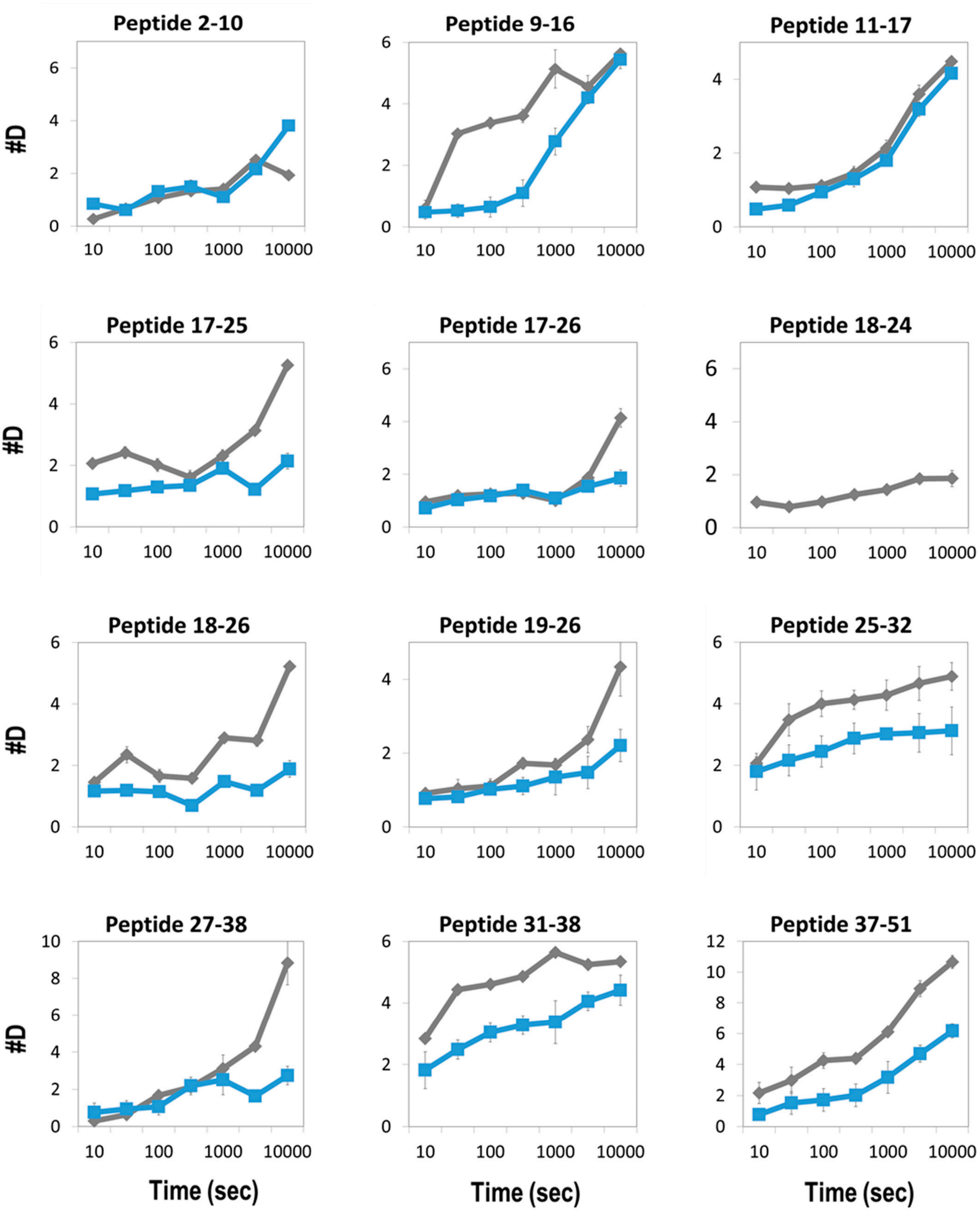
Peptide 2-10
Peptide 9-16
Peptide 11-17
Peptide 17-25
Peptide 17-26
Peptide 18-24
Peptide 18-26
Peptide 19-26
Peptide 25-32
Peptide 27-38
Peptide 31-38
Peptide 37-51
#D
Time (sec)
10
100
1000
10000

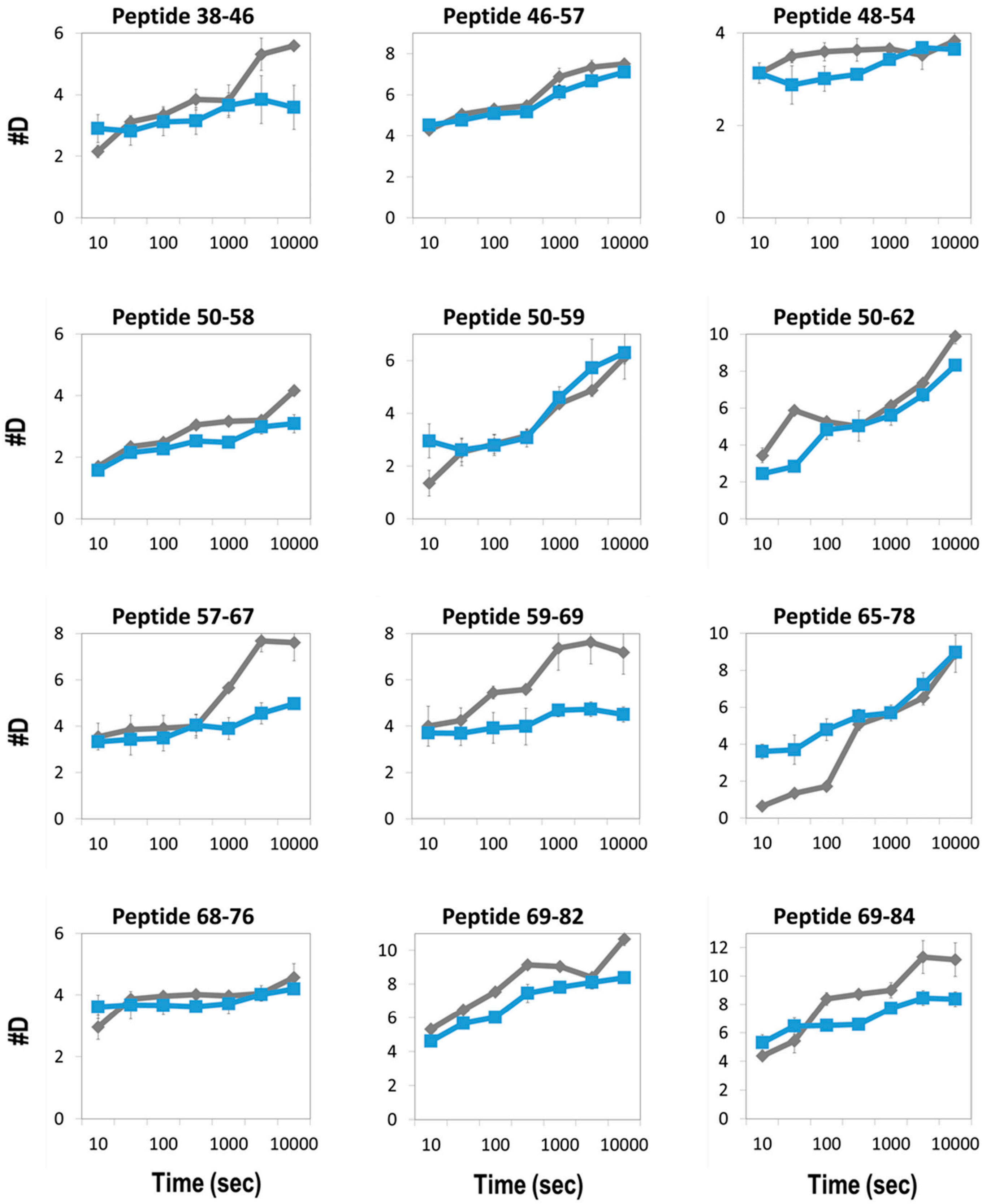

Peptide 38-46
Peptide 46-57
Peptide 48-54
Peptide 50-58
Peptide 50-59
Peptide 50-62
Peptide 57-67
Peptide 59-69
Peptide 65-78
Peptide 68-76
Peptide 69-82
Peptide 69-84
#D
#D
#D
#D
10
100
1000
10000
Time (sec)
Time (sec)
Time (sec)

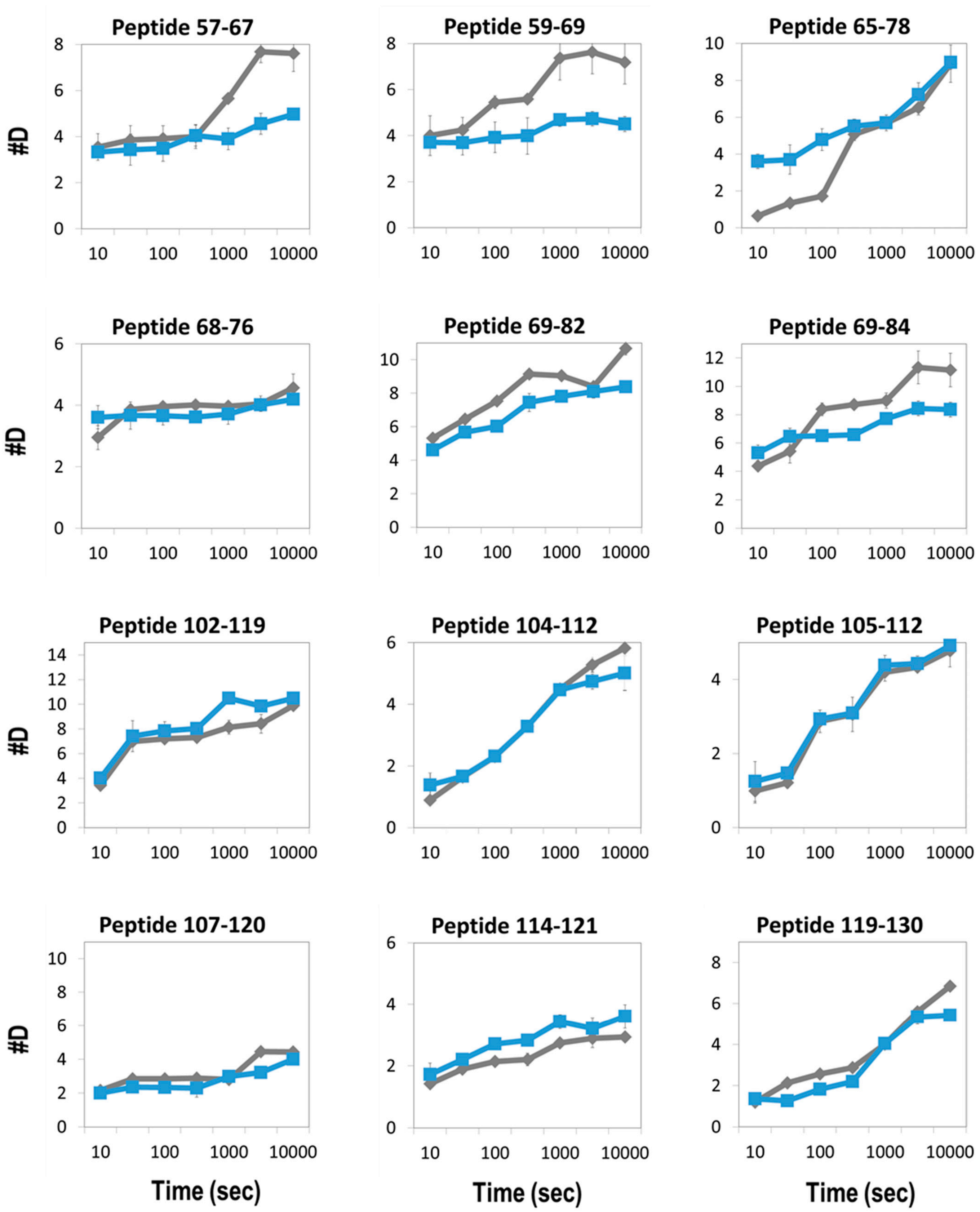

Peptide 57-67
Peptide 59-69
Peptide 65-78
Peptide 68-76
Peptide 69-82
Peptide 69-84
Peptide 102-119
Peptide 104-112
Peptide 105-112
Peptide 107-120
Peptide 114-121
Peptide 119-130
#D
Time (sec)

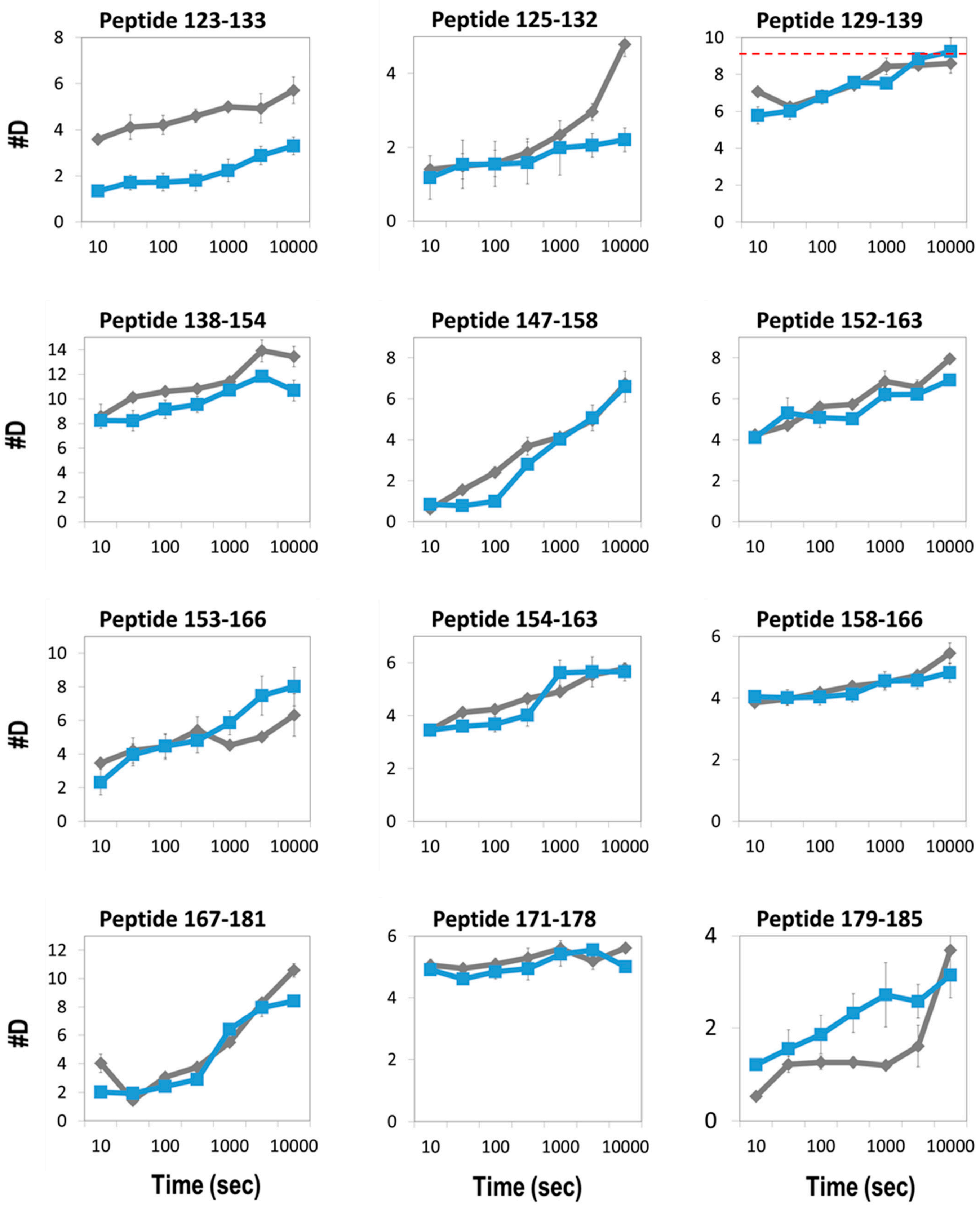
Peptide 123-133
Peptide 125-132
Peptide 129-139
Peptide 138-154
Peptide 147-158
Peptide 152-163
Peptide 153-166
Peptide 154-163
Peptide 158-166
Peptide 167-181
Peptide 171-178
Peptide 179-185
#D
Time (sec)

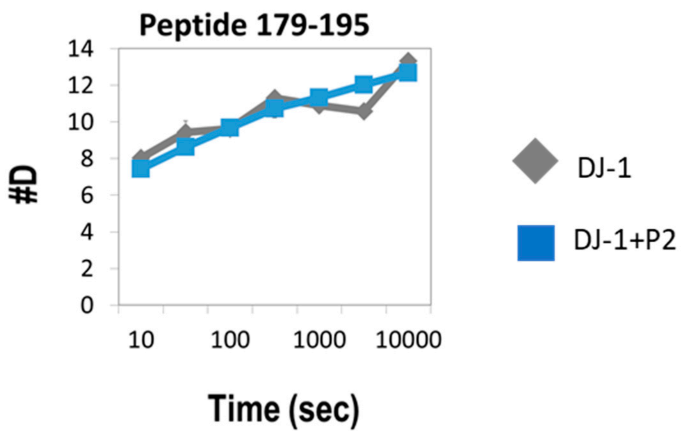

Supplemental Figure S4. H/D exchange results of DJ-1 and peptide 2 bound DJ-1 after back-exchange correction.

DJ-1 bound to P2 was deuterated for 10, 30, 100, 300, 1000, 3000, and 10 000 s. The number of deuteration were adjusted based on the 24-hour back exchange. The maximum value of the Y-axis or at the red dash line is the maximum deuteration number of the peptides. All experiments were conducted in triplicate, and the errors represent the standard deviation. The signal of peptide 18-24 overlapped with P2 and therefore the value cannot be quantified.

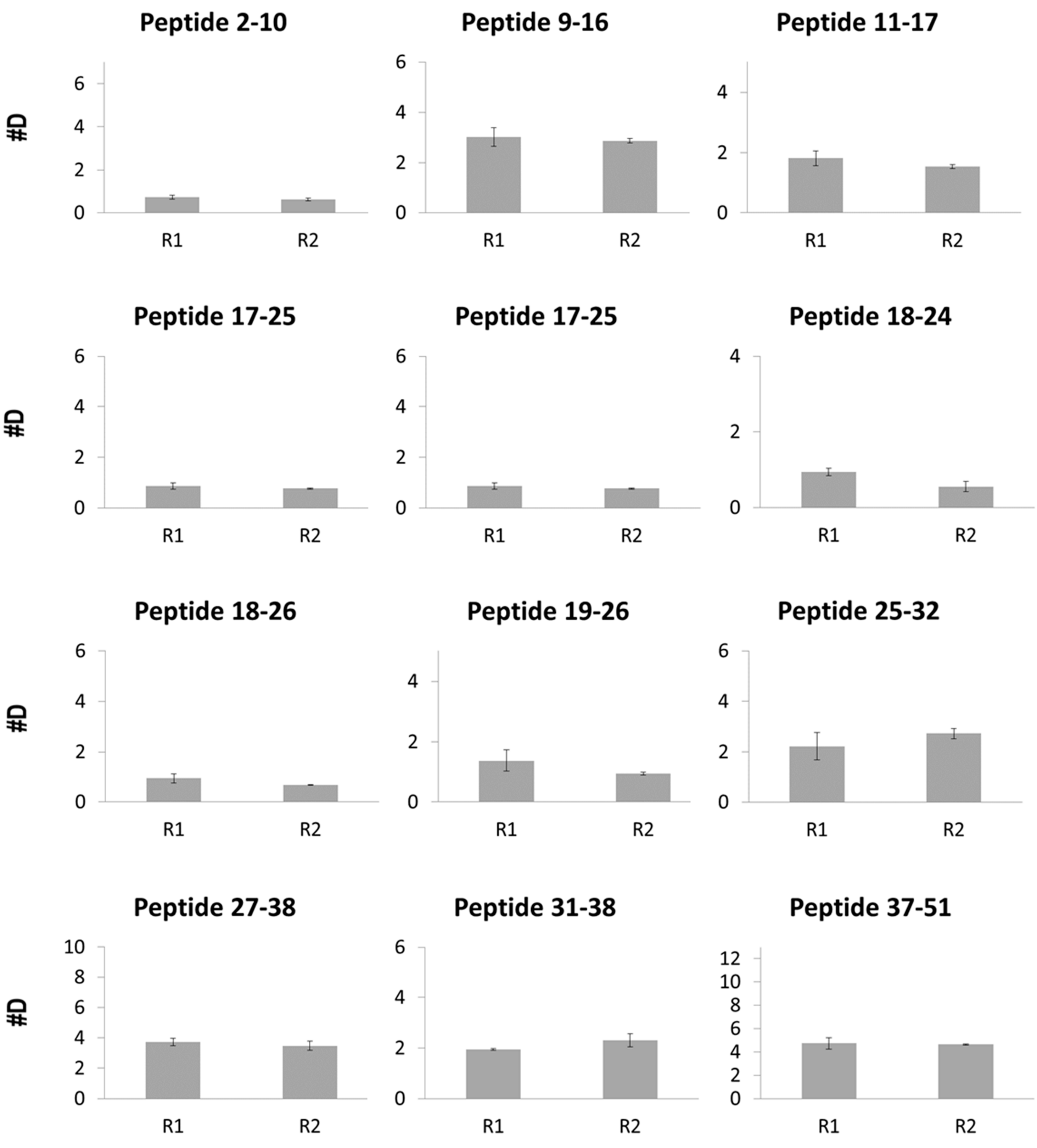
Peptide 2-10
Peptide 9-16
Peptide 11-17
Peptide 17-25
Peptide 17-25
Peptide 18-24
Peptide 18-26
Peptide 19-26
Peptide 25-32
Peptide 27-38
Peptide 31-38
Peptide 37-51
#D
R1
R2

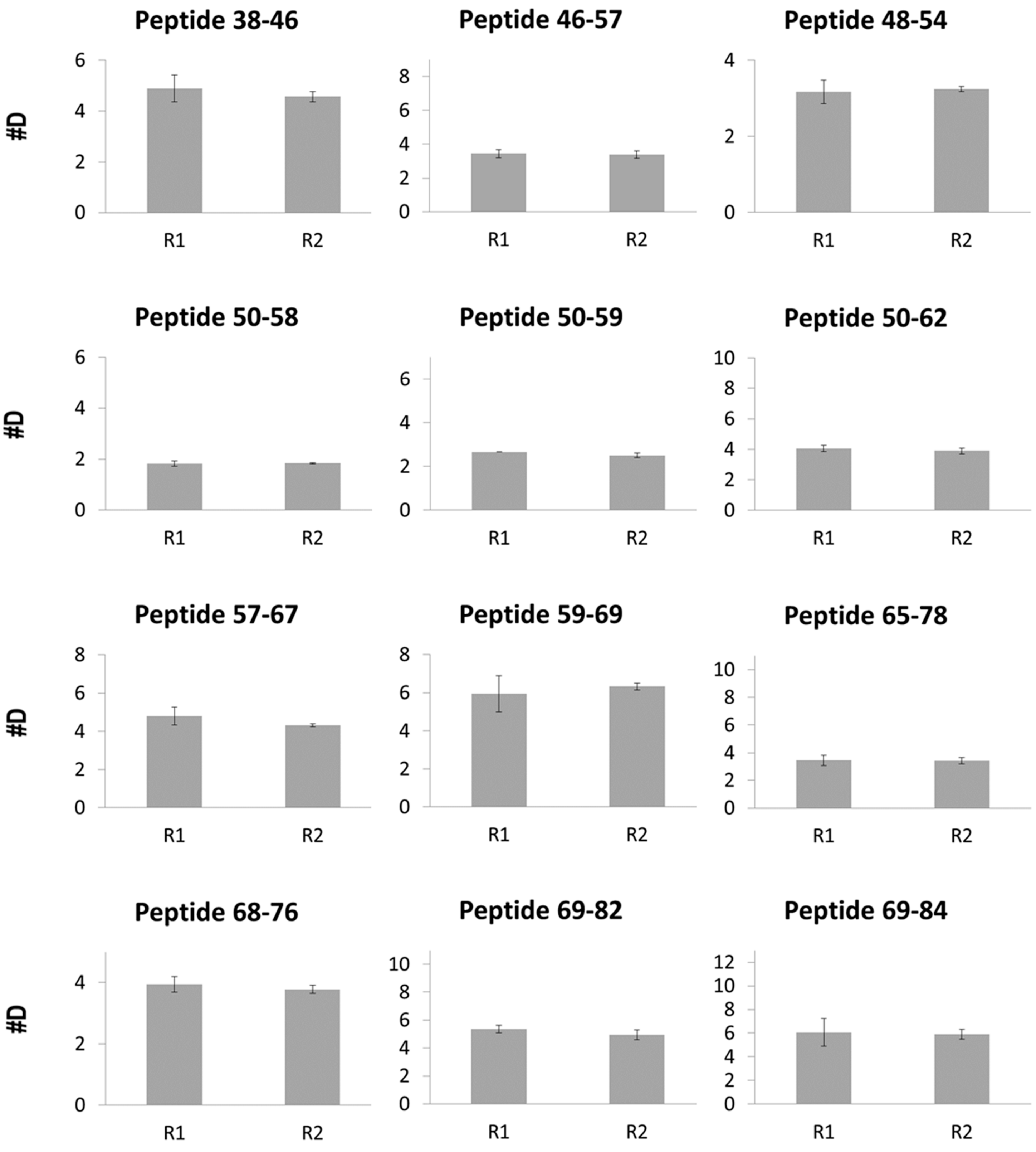
Peptide 38-46
Peptide 46-57
Peptide 48-54
Peptide 50-58
Peptide 50-59
Peptide 50-62
Peptide 57-67
Peptide 59-69
Peptide 65-78
Peptide 68-76
Peptide 69-82
Peptide 69-84
#D
R1
R2

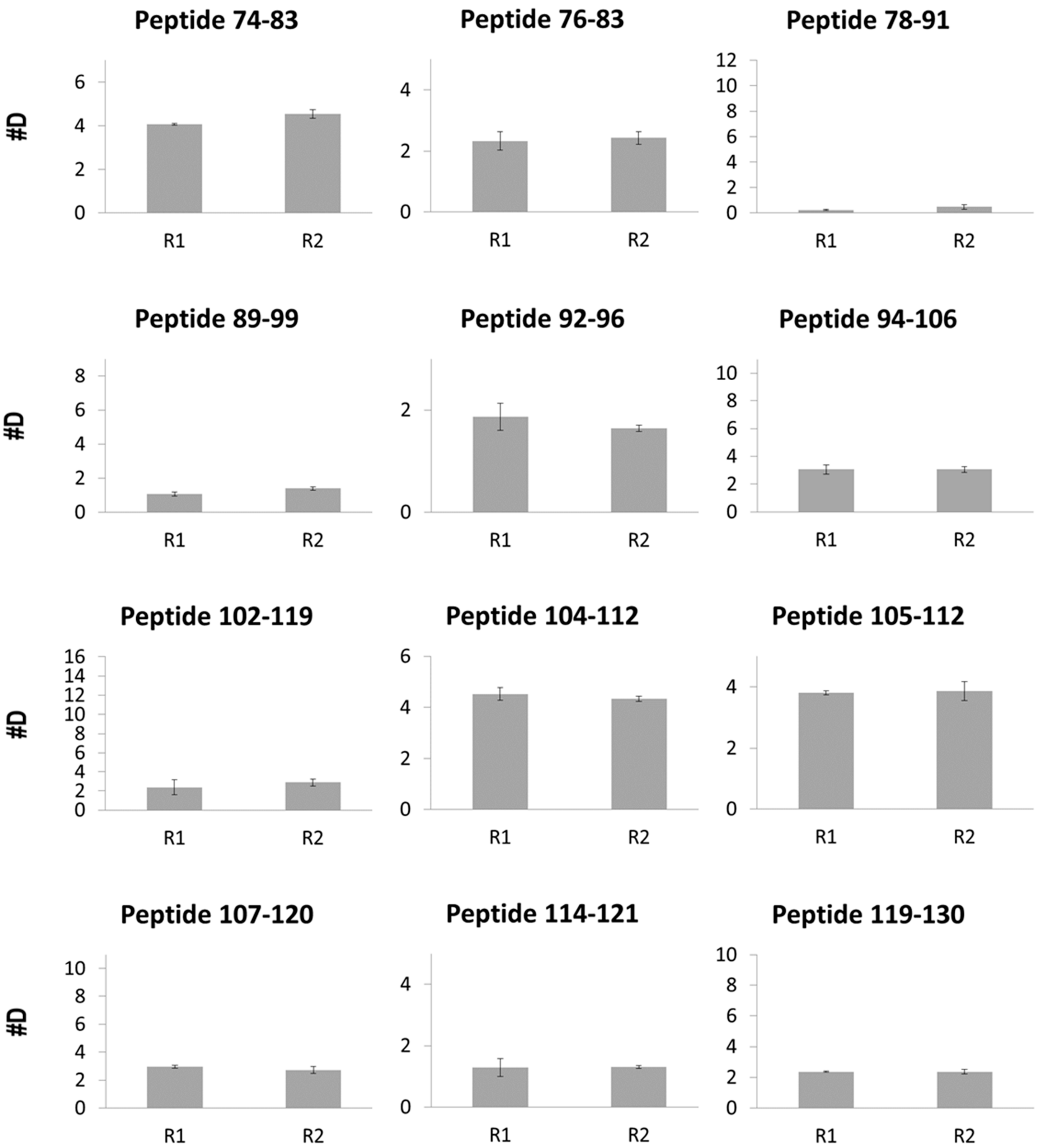

Peptide 74-83
Peptide 76-83
Peptide 78-91
Peptide 89-99
Peptide 92-96
Peptide 94-106
Peptide 102-119
Peptide 104-112
Peptide 105-112
Peptide 107-120
Peptide 114-121
Peptide 119-130
#D
R1
R2

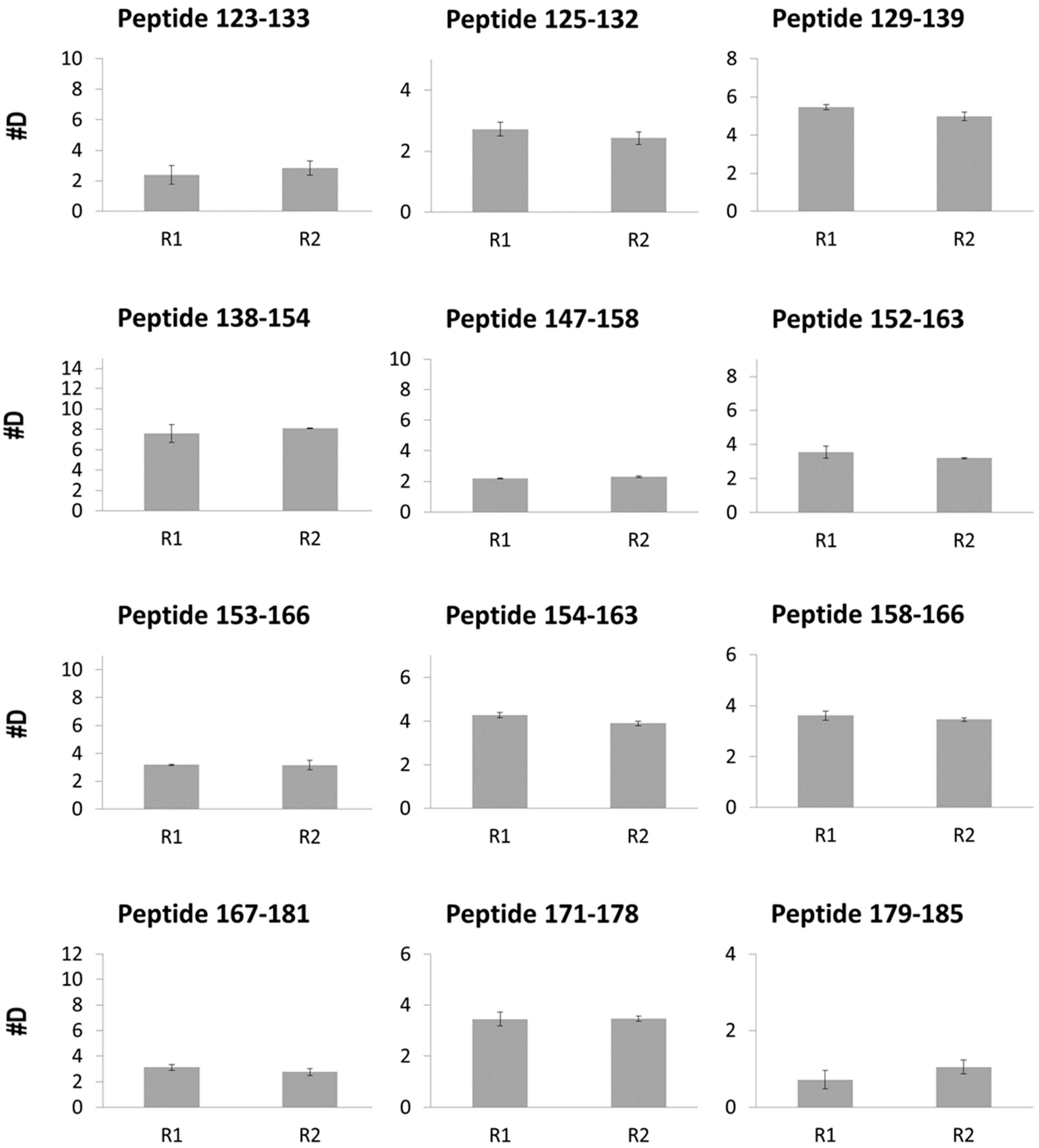
Peptide 123-133
Peptide 125-132
Peptide 129-139
Peptide 138-154
Peptide 147-158
Peptide 152-163
Peptide 153-166
Peptide 154-163
Peptide 158-166
Peptide 167-181
Peptide 171-178
Peptide 179-185
#D
R1
R2

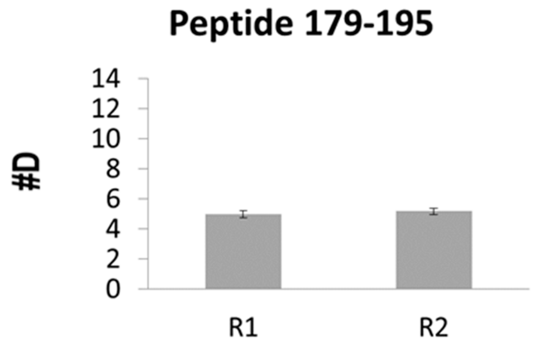

Supplemental Figure S5. Confirmation of the effectiveness of DTT reduction.

The reduction reactions of two protein samples stored in the freezer for four months apart were conducted at two separate time. The HDXMS results in triplicate at 10000 seconds are shown.

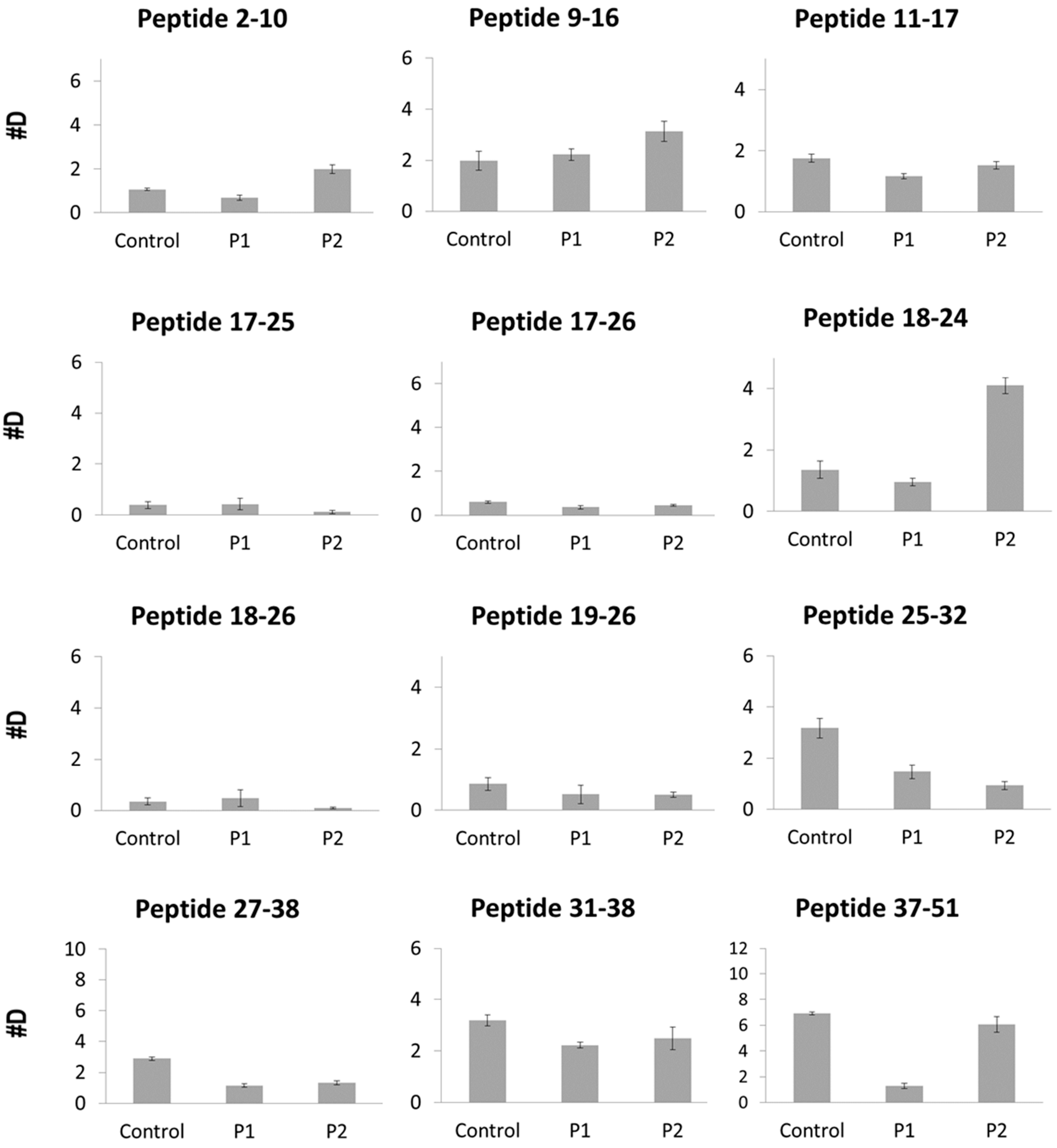

Peptide 2-10
Peptide 9-16
Peptide 11-17
Peptide 17-25
Peptide 17-26
Peptide 18-24
Peptide 18-26
Peptide 19-26
Peptide 25-32
Peptide 27-38
Peptide 31-38
Peptide 37-51
#D
Control
P1
P2

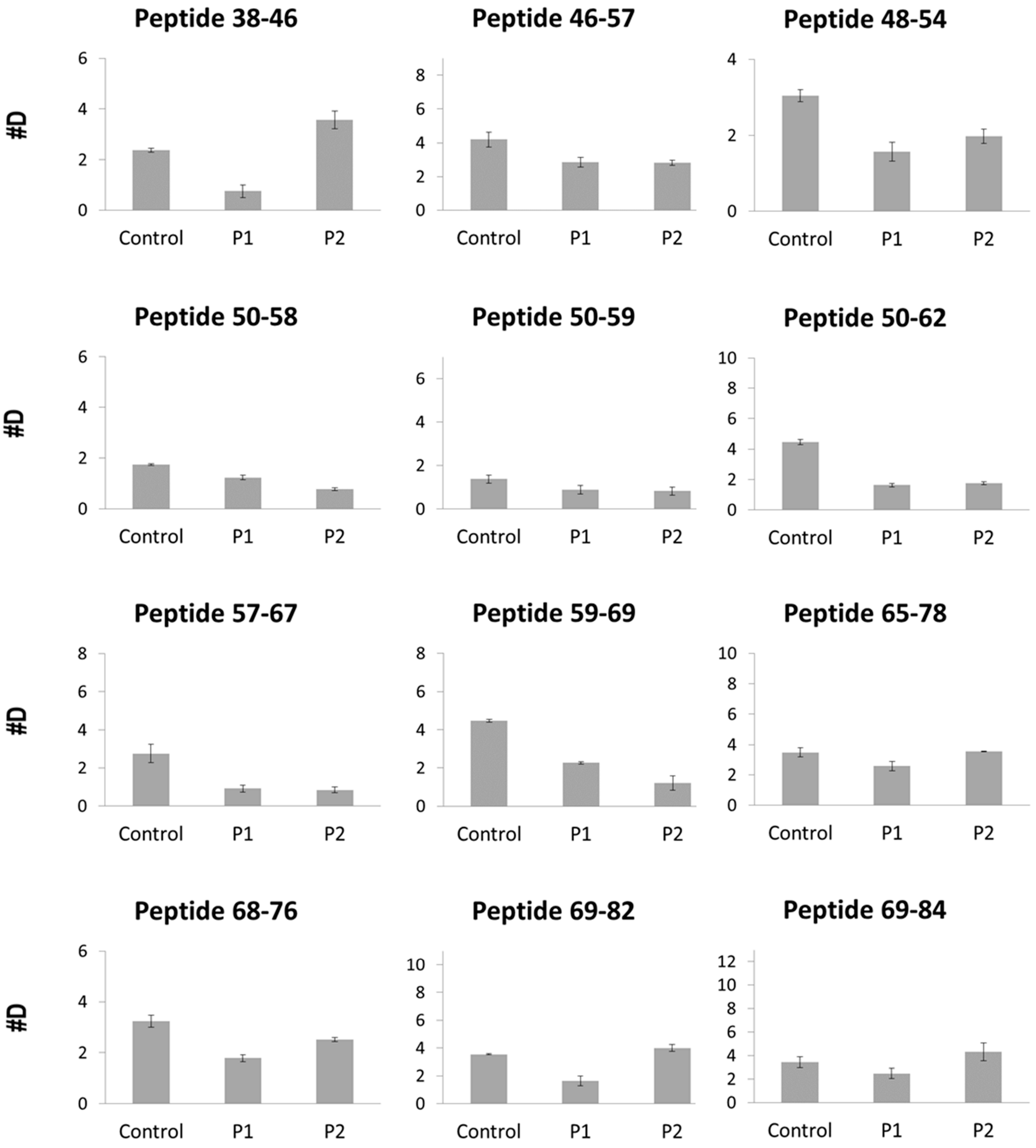

Peptide 38-46
Peptide 46-57
Peptide 48-54
Peptide 50-58
Peptide 50-59
Peptide 50-62
Peptide 57-67
Peptide 59-69
Peptide 65-78
Peptide 68-76
Peptide 69-82
Peptide 69-84
#D
#D
#D
#D
0
2
4
6
8
10
12
Control
P1
P2

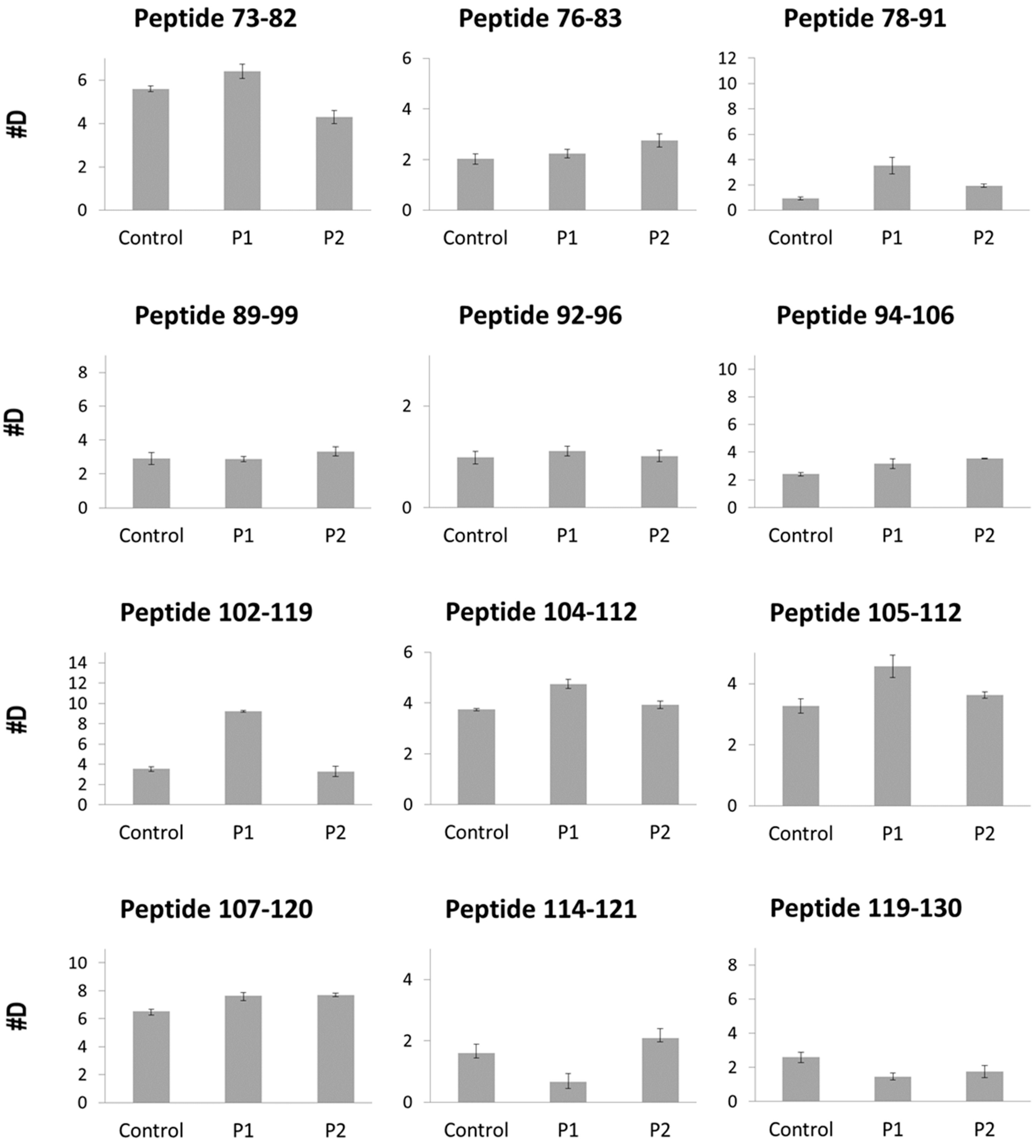
Peptide 73-82
Peptide 76-83
Peptide 78-91
Peptide 89-99
Peptide 92-96
Peptide 94-106
Peptide 102-119
Peptide 104-112
Peptide 105-112
Peptide 107-120
Peptide 114-121
Peptide 119-130
#D
Control
P1
P2

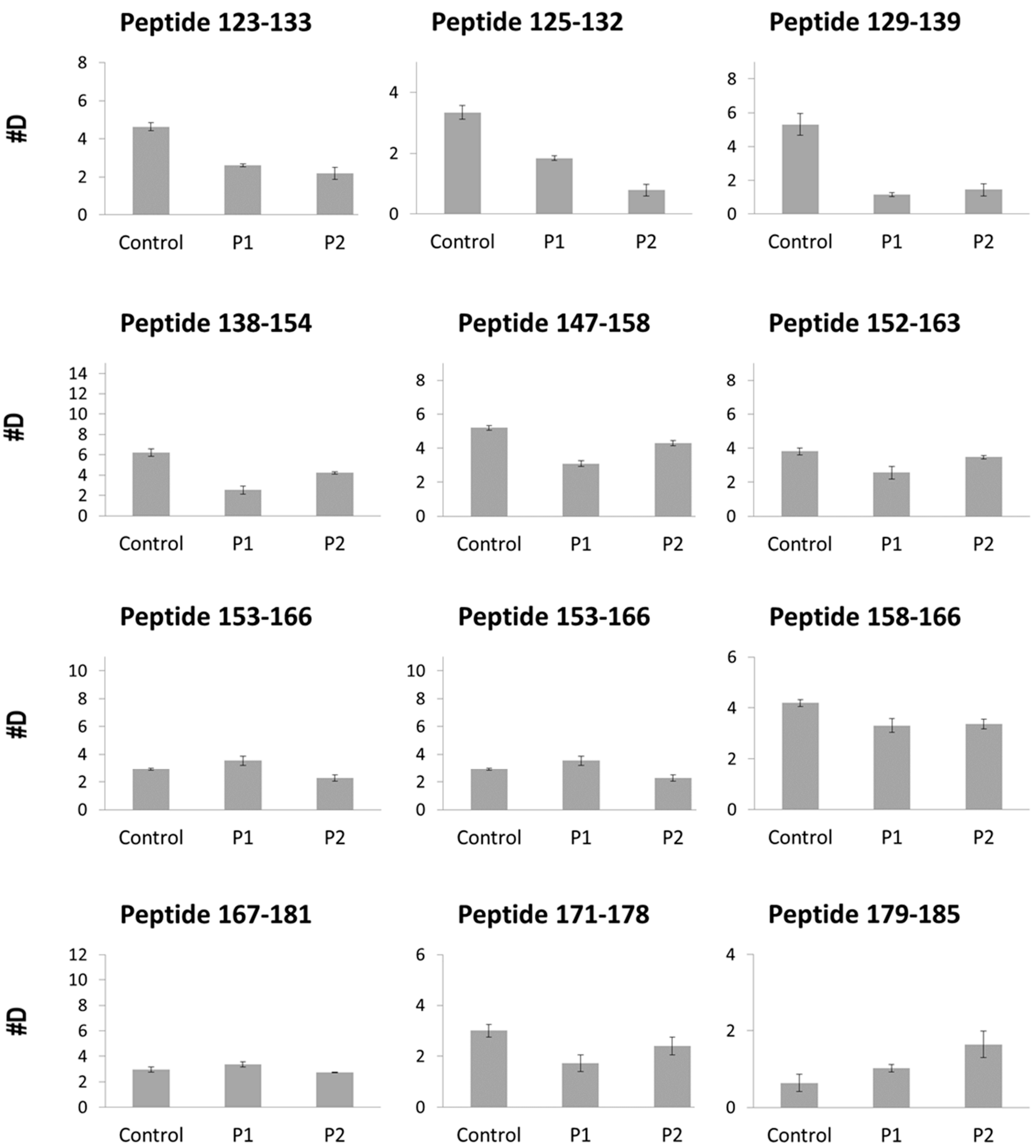

Peptide 123-133
Peptide 125-132
Peptide 129-139
#D
8
6
4
2
0
Control
P1
P2
Peptide 138-154
Peptide 147-158
Peptide 152-163
#D
14
12
10
8
6
4
2
0
Control
P1
P2
Peptide 153-166
Peptide 153-166
Peptide 158-166
#D
10
8
6
4
2
0
Control
P1
P2
Peptide 167-181
Peptide 171-178
Peptide 179-185
#D
12
10
8
6
4
2
0
Control
P1
P2

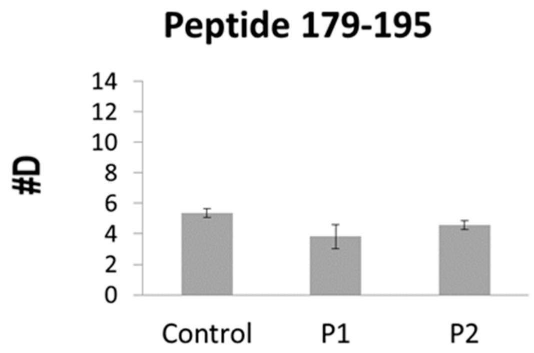

Supplemental Figure S6. HDXMS results of DJ-1 and P1 and P2 bound DJ-1.
An analysis of the variation in H/D exchange at 10000 sec through the addition of P1 and P2 to the originally reduced DJ-1 protein without DTT treatment.

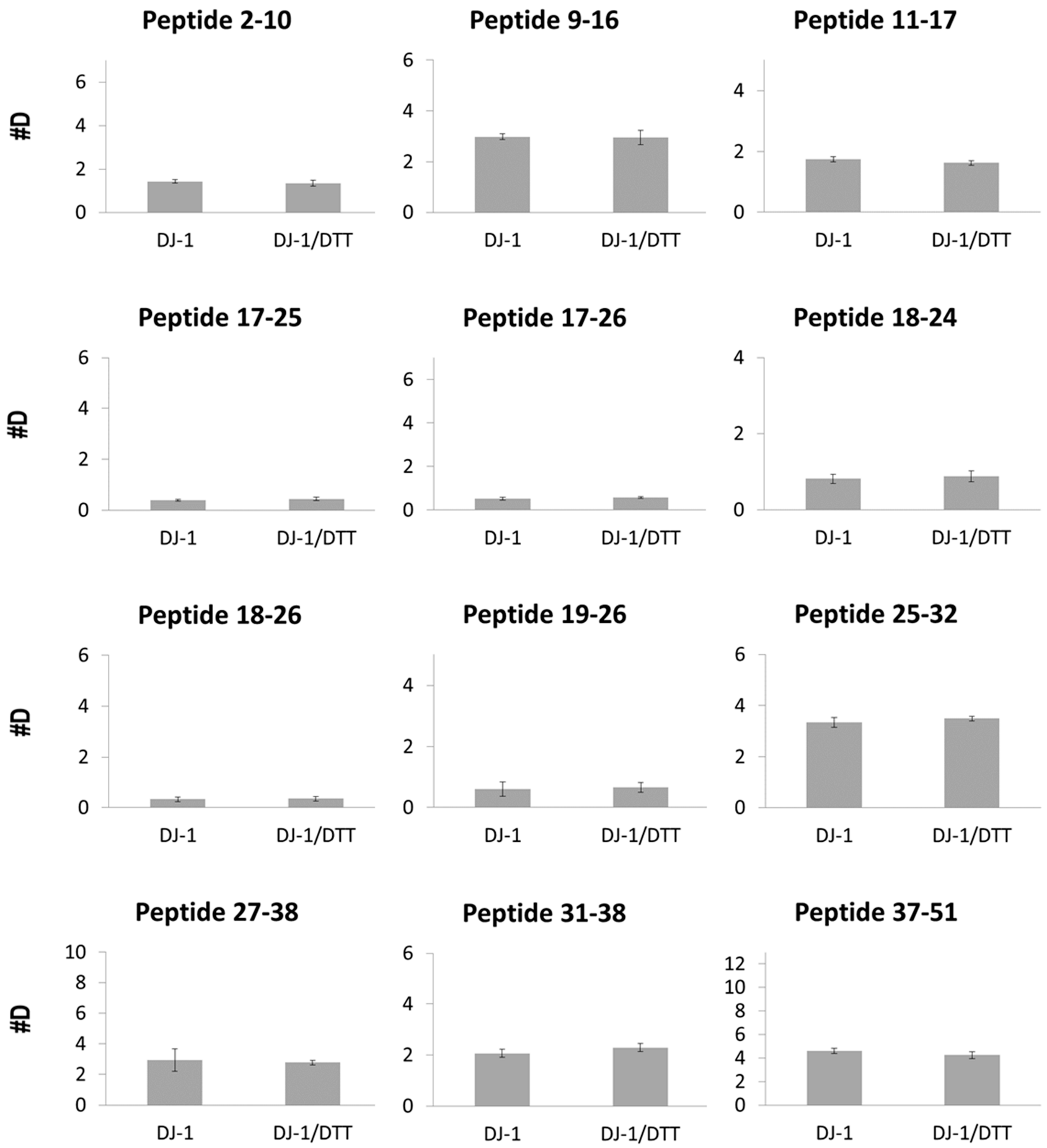
Peptide 2-10
Peptide 9-16
Peptide 11-17
Peptide 17-25
Peptide 17-26
Peptide 18-24
Peptide 18-26
Peptide 19-26
Peptide 25-32
Peptide 27-38
Peptide 31-38
Peptide 37-51
#D
DJ-1
DJ-1/DTT

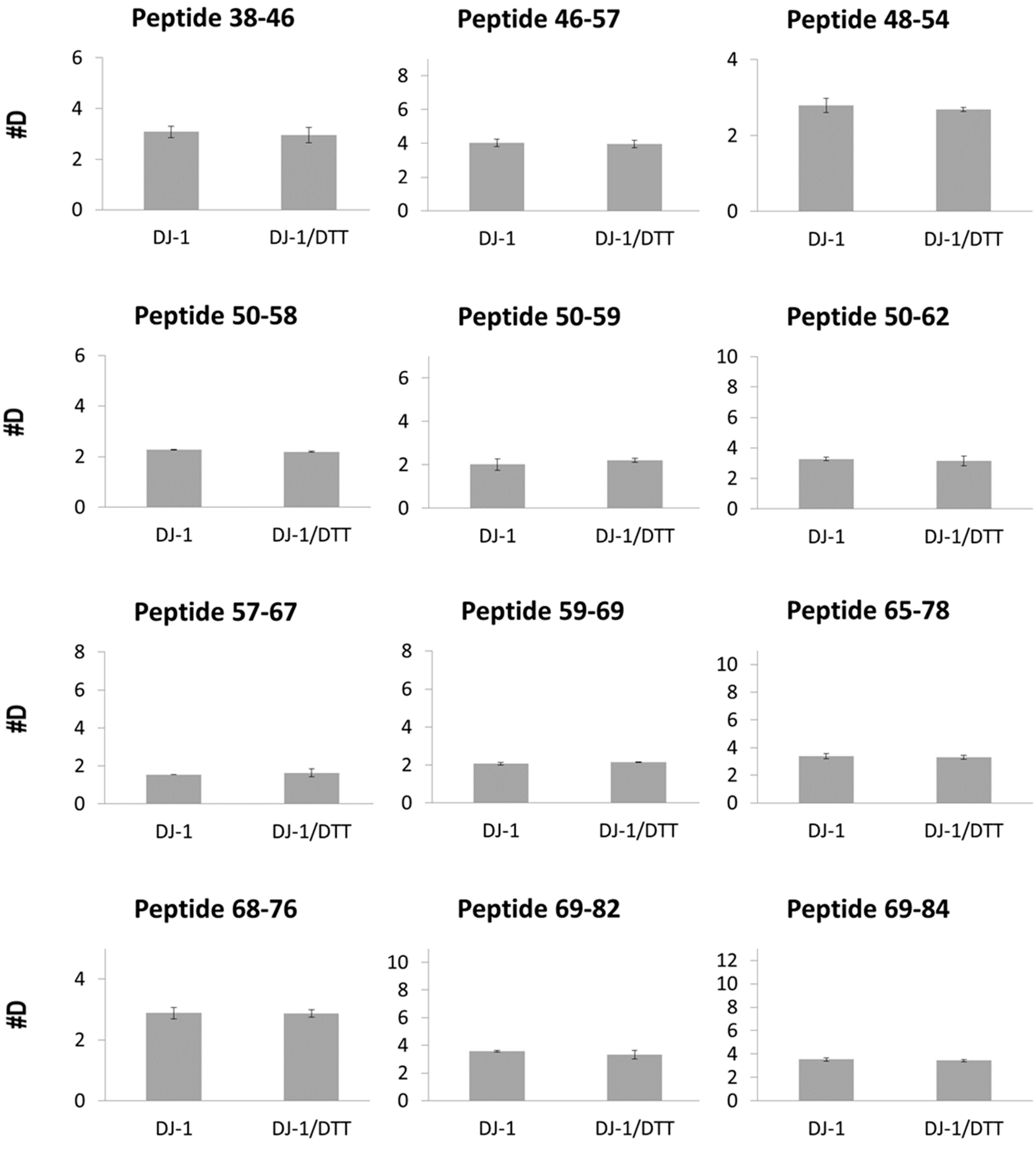
Peptide 38-46
Peptide 46-57
Peptide 48-54
Peptide 50-58
Peptide 50-59
Peptide 50-62
Peptide 57-67
Peptide 59-69
Peptide 65-78
Peptide 68-76
Peptide 69-82
Peptide 69-84
#D
DJ-1
DJ-1/DTT

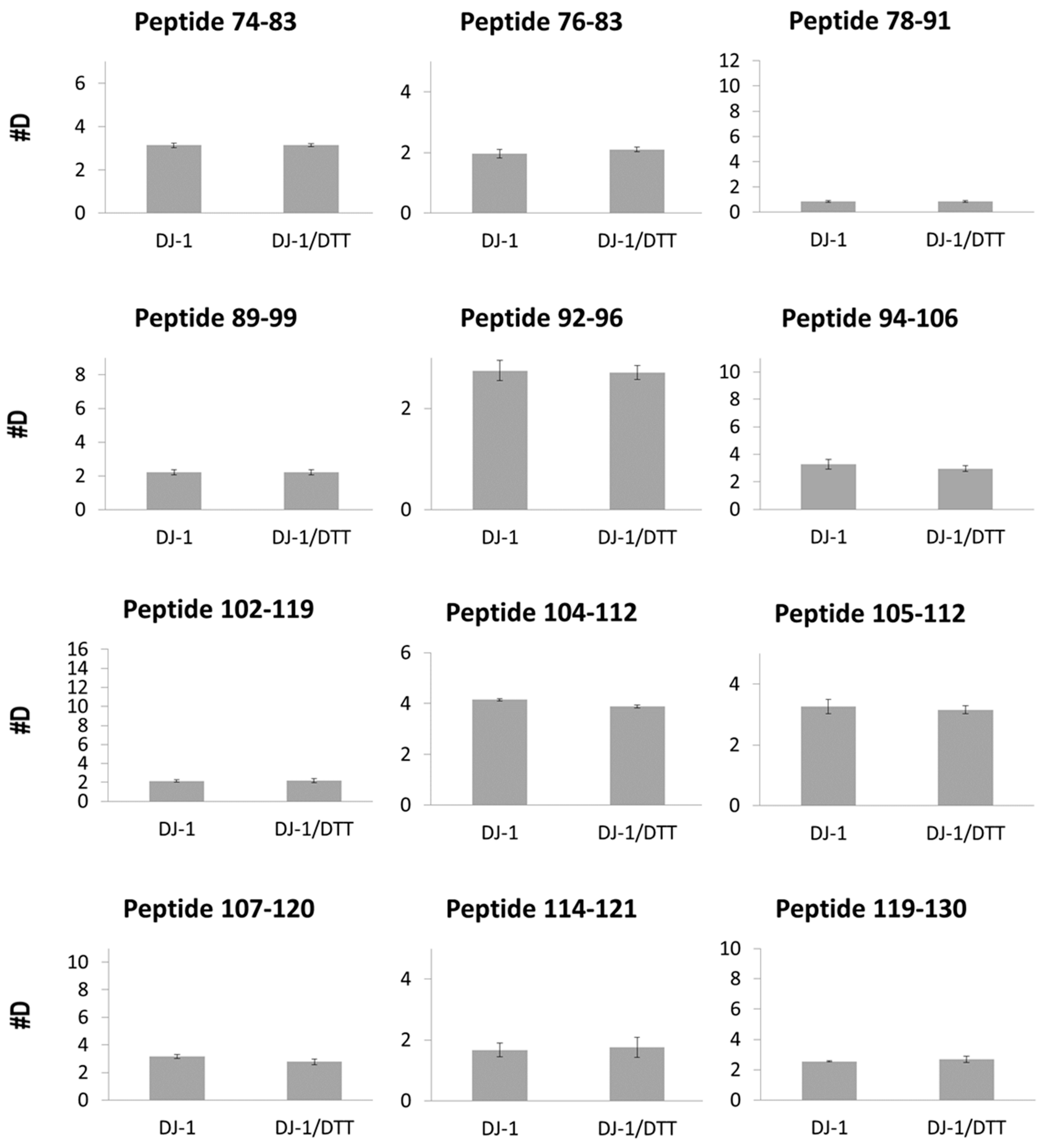

Peptide 74-83
Peptide 76-83
Peptide 78-91
Peptide 89-99
Peptide 92-96
Peptide 94-106
Peptide 102-119
Peptide 104-112
Peptide 105-112
Peptide 107-120
Peptide 114-121
Peptide 119-130
#D
DJ-1
DJ-1/DTT

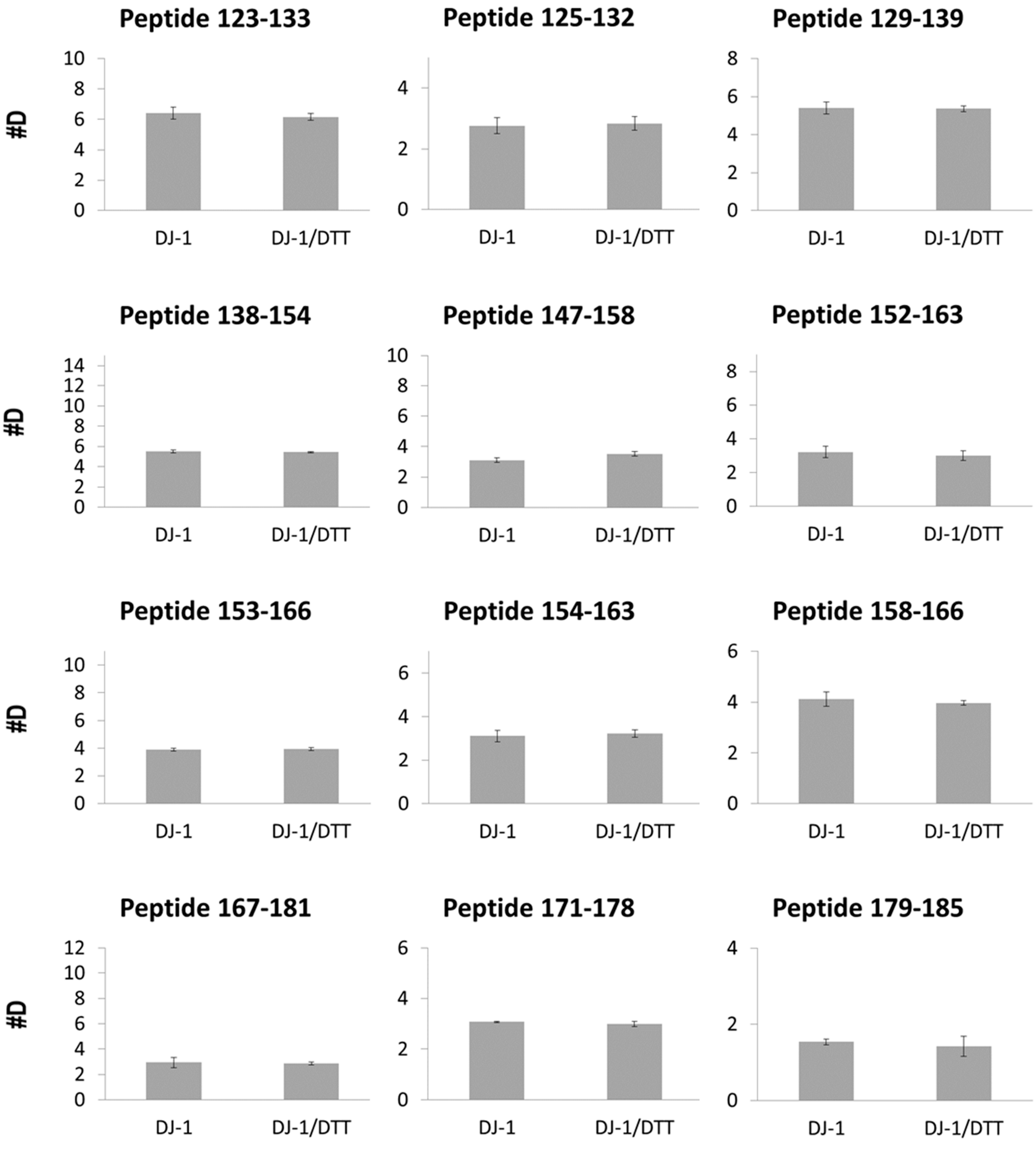
Peptide 123-133
Peptide 125-132
Peptide 129-139
Peptide 138-154
Peptide 147-158
Peptide 152-163
Peptide 153-166
Peptide 154-163
Peptide 158-166
Peptide 167-181
Peptide 171-178
Peptide 179-185
#D
DJ-1
DJ-1/DTT

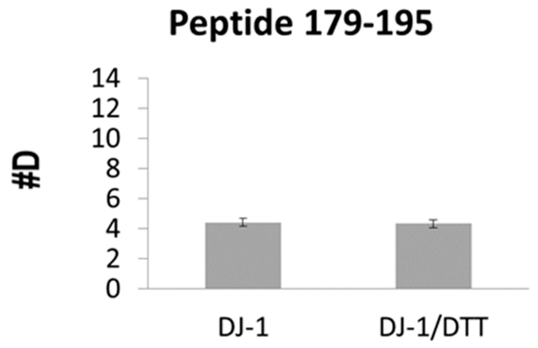

Supplemental Figure S7. The HDXMS results of DTT effects on the originally reduced DJ-1. The protein were in the reduced form naturally while purified from *E. Coli*. The HDXMS experiments at 10000 seconds were conducted on the originally reduced DJ-1. The experiments were conducted in triplicates.
